# Long-term, sustained feeding by Asian citrus psyllid disrupts salicylic acid homeostasis in sweet orange

**DOI:** 10.1186/s12870-019-2114-2

**Published:** 2019-11-12

**Authors:** Freddy Ibanez, Joon Hyuk Suh, Yu Wang, Lukasz L. Stelinski

**Affiliations:** 1 Department of Entomology and Nematology, Citrus Research and Education Center, University of Florida, Lake Alfred, FL 33850 USA; 2 Department of Food Science and Human Nutrition, Citrus Research and Education Center, University of Florida, Lake Alfred, FL 33850 USA

**Keywords:** Salicylic acid, *Diaphorina citri*, Plant defense, Metabolomics, Gene expression, Vector-host interaction, Huanglongbing

## Abstract

**Background:**

Phloem-feeding insects are known to modulate the salicylic acid (SA) signaling pathway in various plant-insect interaction models. *Diaphorina citri* is a phloem feeding vector of the deadly phytopathogens, *Candidatus* Liberibacter americanus and *Candidatus* Liberibacter asiaticus, and the interactions of *D. citri* with its host that may modulate plant defenses are not well understood. The objectives of this study were to investigate the molecular mechanisms involved in transcriptional regulation of SA modification and activation of defense-associated responses in sweet orange (*Citrus sinensis*) exposed to various durations (7-, 14- and 150- days) of continuous feeding by *D. citri*.

**Results:**

We quantified expression of genes involved in SA pathway activation and subsequent modification, as well as, associated SA metabolites (SA methyl ester, 2,3-DHBA, and SA 2-O-β-D-glucoside). *NPR1* and *PR-1* expression was upregulated in plants exposed to continuous feeding by *D. citri* for 14 days. Expression of *BSMT-like*, *MES1-like* and *DMR6-like oxygenase*, as well as, accumulation of their respective SA metabolites (SA methyl ester, 2,3-DHBA) was significantly higher in plants exposed to continuous feeding by *D. citri* for 150 days than in those without *D. citri* infestation. Concomitantly, expression of *UGT74F2-like* was significantly downregulated and its metabolite, SA 2-β-D-glucoside, was highly accumulated in trees exposed to 150 d of feeding compared to control trees without *D. citri*.

**Conclusions:**

*D. citri* herbivory differentially regulated transcription and SA-metabolite accumulation in citrus leaves, depending on duration of insect feeding. Our results suggest that prolonged and uninterrupted exposure (150 d) of citrus to *D. citri* feeding suppressed plant immunity and inhibited growth, which may highlight the importance of vector suppression as part of huanglongbing (HLB) management in citrus.

## Background

Hemipteran, phloem-feeding insects have been categorized into three main suborders: Sternorrhyncha (whiteflies, aphids, mealybugs, and psyllids), Auchenorrhyncha (planthoppers, leafhoppers, treehoppers, spittlebugs and cicadas), and Heteroptera (seed bugs, stink bugs, assassin bugs, leaf-footed bugs and bedbugs) [1]. Many hemipterans are economically important as pests of major agricultural crops [2–4]; and vectors of pathogenic microbes of humans, animals, and plants [5, 6]. Within phloem-feeding hemipterans, aphids and whiteflies are the most studied models in plant-insect interactions. However, little is known about interactions between psyllids and their hosts and specifically with regards to plant defense. *Diaphorina citri* Kuwayama (Hemiptera: Lividae), commonly known as Asian citrus psyllid (ACP), is a phloem-feeding insect of citrus species that transmits phytopathogens, *Candidatus* Liberibacter spp., causing huanglongbing (HLB), also known as citrus greening. This plant disease can devastate citrus production in affected areas worldwide. Since HLB was detected in Florida in 2005, citrus production has progressively declined resulting in $4.55 billion in lost revenue and > 8000 job losses since 2011 [7]. Therefore, deeper understanding of the interaction between insect (vector) and plant (host) is critical to develop novel management strategies.

Phloem feeders rely on their highly modified mouthparts (stylets) to navigate through various plant cell layers to reach specific feeding sites [8]. In particular, *D. citri* adults ingest mostly from the phloem sieve elements, and to a lesser extent they also appear to ingest sap associated with xylem vessels [9–11]. During phloem ingestion, a number of salivary effector proteins have been identified in aphids, planthoppers, and spider mites. These effectors modulate several processes such as, fecundity, host colonization, and modulation of plant-defense signaling pathways, as reviewed in Kaloshian and Walling [12] and Xu, Qian [13]. To date, homologous salivary effectors have not been identified within the *D. citri* genome. But, it is known that *D. citri* feeding on citrus triggers the release of methyl salicylate (MeSA), [14], implicating the SA pathway in the *Citrus sinensis - D. citri* interaction.

Salicylic acid (SA) plays a crucial role in plant innate immunity and its synthesis occurs via the isochorismate and phenylalanine ammonia-lyase pathways in plants, both of which have been discussed and reviewed in detail by Chen, Zheng [15] and D’Maris Amick Dempsey, Vlot [16]. Recently, a new mechanism for SA accumulation was described in *Arabidopsis thaliana*, in which avrPphB Susceptible 3 (PBS3) in the cytosol catalyzes the formation of Isochorismate-9-glutamate (ISC-9-Glu), which spontaneously decays into SA without the requirement of an enzymatic catalysis [17]. Once SA is biosynthesized, several chemical modifications occur within cells, including methylation, glycosylation, hydrolyzation and amino acid conjugations. The methylation process, represented by MeSA, is synthetized by a benzoic acid/salicylic acid carboxyl methyltransferase (BSMT) enzyme [18]. Esterification of MeSA is mediated by SA-binding protein 2 (Sabp2) and methylesterases, which transform MeSA into SA [19–21]. The glycosylate molecule of SA, SA 2-β-D-glucoside, is catalyzed by UDP-glucosyltransferases (UGT74F1 and UGT74F2) [22–24]. Also, SA can be subsequently hydroxylated to generate the molecules 2,3-dihydroxybenzoic acid (2,3-DHBA) and 2,5-dihydroxybenzoic acid (2,5-DHBA) [25, 26]. The SA metabolites are inactive forms of SA; however, they may have some biological functions. It has been suggested that MeSA is associated with triggering of systemic acquired resistance (SAR) in Arabidopsis, tobacco, and potato in response to microbial pathogens [19–21, 27], and causes indirect defense by attraction of natural enemies [28–30]. Application of SA 2-β-D-glucoside in tobacco leaves induces expression of the SA marker gene, *Pathogenesis related-1 (PR-1)* [31]. Hydroxylated SA molecules induce different plant responses; for example, the application of 2,3-DHBA induced weak *PR-1* expression as compared with applications of SA [32]. However, 2,5-DHBA induced the synthesis of a different set of PR proteins [33].

**Fig. 1:**
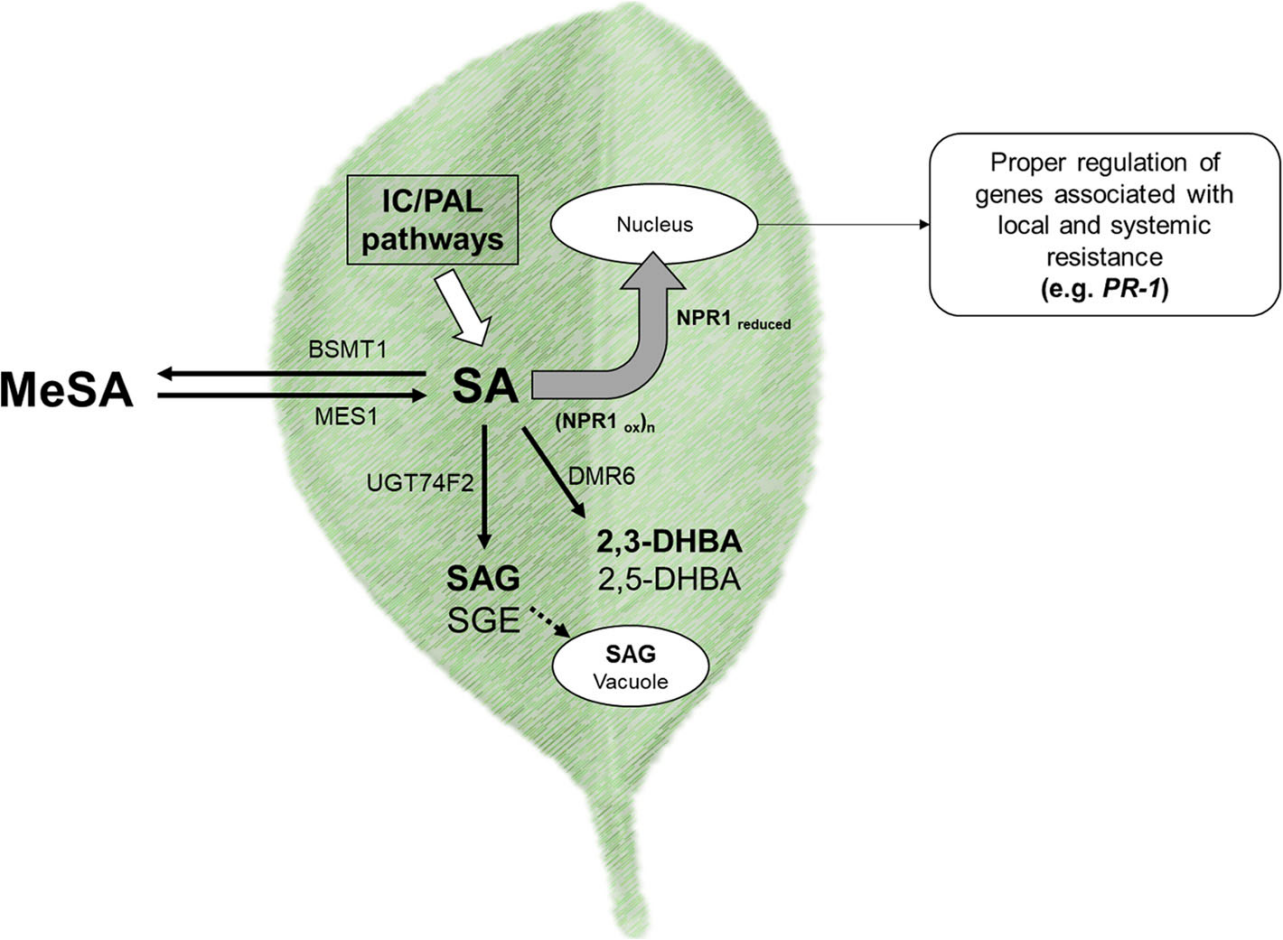
Working model of the SA-dependent pathway and SA modifications in *Citrus sinensis*. Proposed (simplified) model of the molecular mechanisms involved in the transcriptional regulation of SA modification and activation of defense-associated responses via NPR1 in *C. sinensis*. Black line arrows indicate the synthesis of SA metabolites. Dashed line indicates transport to vacuole; thick, white arrow indicates the Isochorismate (IC) Phenylalanine ammonia-lyase pathways; and thick, gray arrow indicates the reduction and monomerization of NPR1. The abbreviations are as follows: isochorismate (IC) and phenylalanine ammonia-lyase (PAL) pathways. Molecules are abbreviated as follows: salicylic acid (SA), methyl salicylate (MeSA), SA 2-β-D-glucoside (SAG), salicylate glucose ester (SGE), 2,3-dihydroxybenzoic acid (2,3-DHBA) and 2,5-dihydroxybenzoic acid (2,5-DHBA). Proteins are abbreviated as: benzoic acid/ salicylic acid carboxyl methyltransferase 1 (BSMT1), Methylesterase 1 (MES1), Downy mildew resistance 6 (DMR6), UDP-glucosyltransferase 74F2 (UGT74F2), Nonexpresser of Pathogenesis-Related genes 1 oxidized or reduced forms (NPR1ox/NPR1reduced), Pathogenesis-related 1 gene (PR-1)

During downstream SA signaling, various proteins have been described. For example, the transcription cofactor NONEXPRESSER OF PATHOGENESIS RELATED GENES 1 (NPR1) occurs. NPR1 contains two conserved protein–protein interaction domains: the BTB (Bric-a-brac, Tramtrack, Broad-complex) domain and the ankyrin repeat domain. NPR1 interacts with TGA transcription factors [34], and has been proposed to function as a transcription co-activator of systemic acquired resistance (SAR) gene expression [35, 36]. NPR1 acts by inducing the expression of pathogenesis related (PR) genes, but also downregulates the expression of genes involved in basic cellular processes, which for example decrease plant growth [35, 37] (Fig. 1).

Deployment of SA-dependent immune responses is associated with SA accumulation *in planta*. Understanding the mechanism(s) of immune response in *C. sinensis* via accumulation of SA and its metabolites after prolonged feeding by uninfected *D. citri* may help address basic questions related to HLB disease management. For example, the continued importance of vector suppression in areas where HLB disease is endemic is unknown. Given the cost of agrochemicals and their potential environmental impact, the need for vector management in areas where nearly 100% of trees are infected has been a controversial subject [38].

In this study, we hypothesized that the SA-dependent pathway and SA metabolism are differentially induced in *Citrus sinensis* after various degrees of injury inflicted by *D. citri* feeding (short versus long-term). We describe transcriptional regulation of genes involved in SA pathway activation and subsequent modification, as well as, their associated metabolites in *C. sinensis* challenged to *D. citri* for various durations of feeding. An overarching goal is discovery of potential targets associated with SA modifications that could manipulate plant defense in citrus to mitigate decline caused by HLB.

## Results

### Identification of *Citrus sinensis* homologous genes

The selection of genes was achieved by analyzing the percentage of nucleotide identity and qPCR primer-specificity. In particular, for *BSMT-like*, three homologs were identified (accession numbers: XM_006466773, XM_006471131 and XM_006470290). Three sets of qPCR primers were tested in cDNA samples, but only one *BSMT-like* transcript (accession number XM_006466773) showed a unique amplicon; therefore, we discarded the other two *BSMT-like* homologs (XM_006471131 and XM_006470290) from further analysis. In the case of *DMR6-like oxygenase,* three homologs were determined in *C. sinensis* (accession numbers: KK784903, XM_015533952 and XM_006465249). Among these three *DMR6-like* homologs, the percentage of nucleotide identity (~ 92%) did not allow us to find a set of qPCR primers that could differentiate each homolog. For the transcripts, *UGT74F2-like* (accession number XM_006478492) and *MES1-like* (accession number XM_015532488), one additional homolog was identified for each (accession numbers: XM_006478493 and XM_006485562, respectively) in *C. sinensis.* However, the percentage of nucleotide identity did not allow us to design a set of qPCR primers to differentiate each of these two homologs.

Bayesian analyses were conducted to evaluate the association among the deduced protein sequences of *C. sinensis* with selected homologous proteins from *A. thaliana* (Additional file 1: Figure S1, Additional file 2: Figure S2, Additional file 3: Figure S3 and Additional file 4: Figure S4). The deduced amino acid sequences of BSMT (Accession number XM_006466773) clustered within the clade formed by S-adenosyl-L-methionine-dependent methyltransferases and Salicylate/benzoate carboxyl methyltransferases homologs (Additional file 5: Figure S5). DMR6-like oxygenase (Accession number KK784903) clustered with DMR6-like oxygenases from *C. sinensis* and *A. thaliana* (Additional file 6: Figure S6). MES1 (Accession number XM_015532488) clustered within the clade formed by Salicylic acid binding protein 2 and Methylesterases 1 from *C. sinensis* (Additional file 7: Figure S7). UDP-glycosyltransferases 74 F2 (Accession number XM_006478492) formed a clade with its isoform 2 (Accession number XM_006478493) and clustered with described UDP-glycosyltransferases 74 F1/F2 from *A. thaliana* (Additional file 8: Figure S8). Overall, each selected protein sequence from *C. sinensis* claded within its expected protein family.

The four transcripts associated with SA modifications were identified in the *C. sinensis* genome (BioProject: PRJNA225998). Each Open Reading Frame (ORF) was *in-silico* translated and aligned with a selected homologous proteins from *A. thaliana*, *N. tabacum,* and *Z. mays.* Identical amino acid residues are highlighted in dark-gray boxes. Amino acids sharing similar characteristics of their side chains are displayed in light-gray, while the predicted conserved domains are underlined using round dot lines (Additional file 5: Figure S5, Additional file 6: Figure S6, Additional file 7: Figure S7 and Additional file 8: Figure S8).

Pairwise alignments using *C. sinensis* and *A. thaliana in-silico* translated amino acid (aa) sequences showed the following results: i) DMR6-like oxygenase 1 (346 aa) from *C. sinensis* possessed an identity of 65% and similarity of 80% with a functional salicylic acid 3 hydrolase (348 aa) described in *A. thaliana* (DMR6-like oxygenase 1, Accession number NM_117118) by [25]; ii) a salicylate carboxymethyltransferase (368 aa) from *C. sinensis* possessed an identity of 46% and similarity of 65% with a S-adenosyl-L-methionine-dependent methyltransferase superfamily protein (378 aa) from *A. thaliana* (BSMT1, Accession number NM_111981); iii) a methylesterase1-like (271 aa) from *C. sinensis* possessed an identity of 54% and similarity of 68% to methyl esterase protein from *A. thaliana* (MES1, Accession number NM_127923); and iv) the UDP-glycosyltransferase 74F2-like (454 aa) from *C. sinensis* possessed an identity of 56% and similarity of 73% to its homologous protein (445 aa) in *A. thaliana* (UGT74F2, Accession number NM_129944).

### Expression patterns of genes involved in SA metabolism in leaves of *Citrus sinensis*

**Fig. 2:**
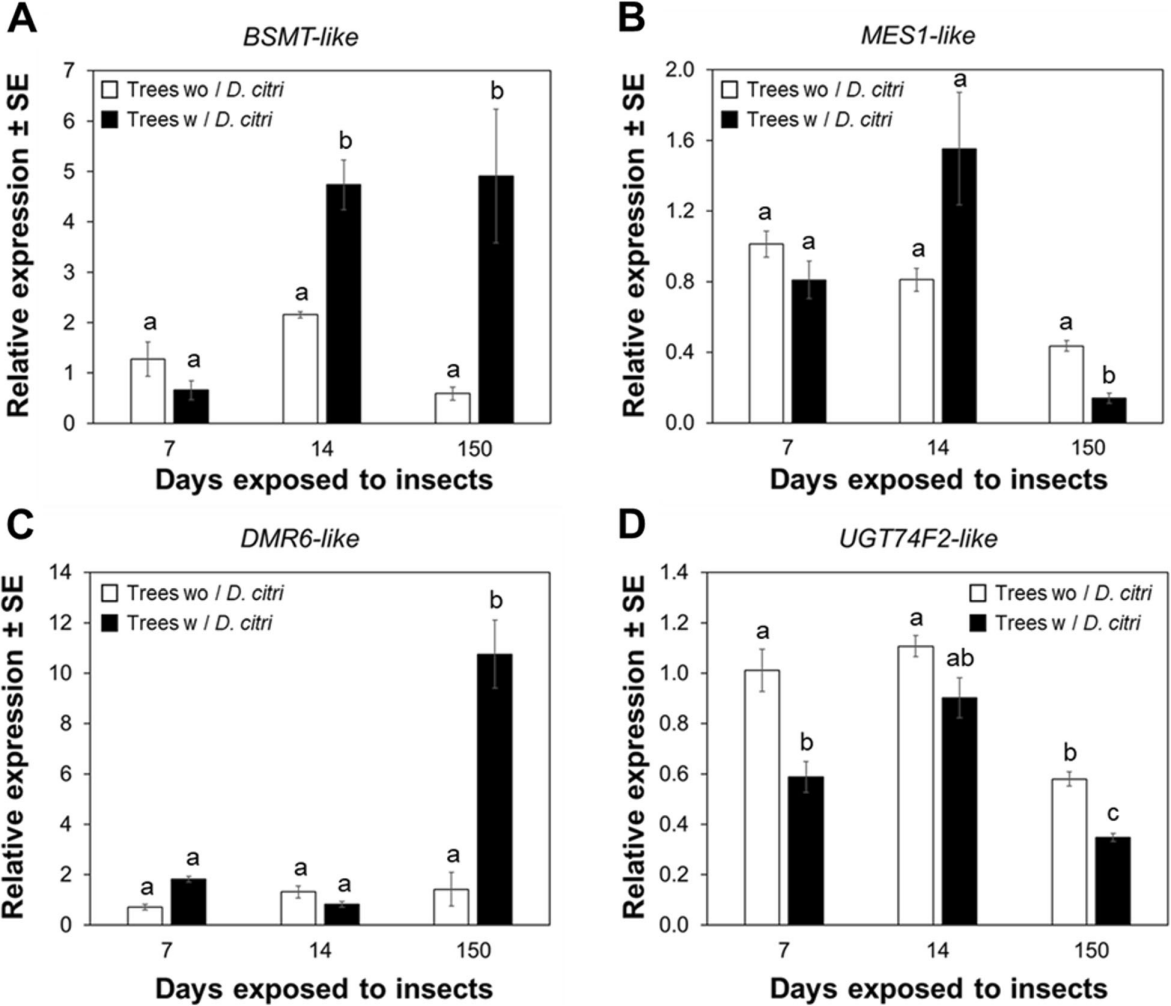
Feeding-induced expression pattern of *BSMT-like, MES1-like, S3H-like,* and *UGT74F2-like* in mature leaves of *Citrus sinensis*. **a** Relative expression of *BSMT-like*. **b** Relative expression of *MES1-like*. **c** Relative expression of *DMR6-like oxygenase*. **d** Relative expression of *UGT74F2-like*. Expression levels were normalized using two references genes, *β-Actin* (accession number XM_026823249.1) and *Elongation factor 1α* (accession number XM_006488084)*.* Data represent mean ± standard deviation (SD) of six biological replicates per treatment. Different letters indicate statistical differences between treatments at *P* < 0.05 using one-way ANOVA with Tukey’s post hoc test

Quantitative RT-PCR (qRT-PCR) analysis showed that the relative expression of *BSMT-like* was significantly (*P* < 0.05) upregulated in plants exposed to continuous feeding by *D. citri* for 14 and 150 d compared with trees without *D. citri* feeding (Fig. 2a). The relative expression of *MES1-like* showed significant (*P* < 0.05) downregulation in plants exposed to continuous feeding by *D. citri* (Fig. 2b). Analysis of *DMR6-like oxygenase* relative expression showed that this transcript was significantly (*P* < 0.05) upregulated in plants exposed to continuous feeding by *D. citri* for 150 d (Fig. 2c). The relative expression of *UGT74F2-like* was significantly (*P* < 0.05) downregulated in trees exposed to *D. citri* for 7 or 150 d compared to control trees without *D. citri* (Fig. 2d).

### Expression patterns of genes involved in SA-dependent pathway in leaves of *Citrus sinensis*

**Fig. 3:**
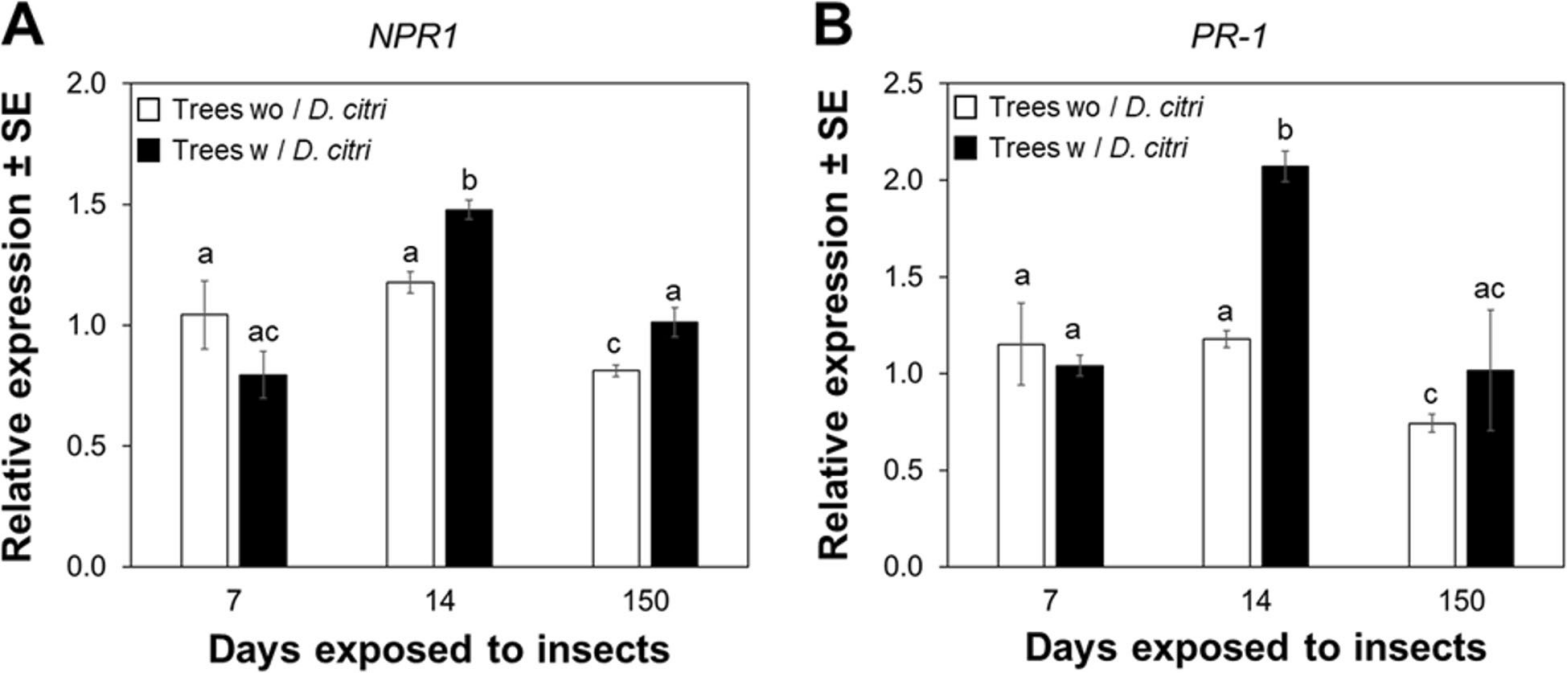
Feeding-induced expression pattern of *NPR1* and *PR-1* in mature leaves of *Citrus sinensis*. **a** Relative expression of *NPR1*. **b** Relative expression of *PR-1*. Expression levels were normalized using two references genes, *β-Actin* (accession number XM_026823249.1) and *Elongation factor 1α* (accession number XM_006488084)*.* Data represent mean ± standard deviation (SD) of six biological replicates per treatment. Different letters indicate statistical differences between treatments at *P* < 0.05 using one-way ANOVA with Tukey’s post hoc test

Quantitative RT-PCR (qRT-PCR) analysis showed that the relative expression of both *NPR1* and *PR-1* was only significantly (*P* < 0.05) upregulated in plants exposed to continuous feeding by *D. citri* for 14 d compared with trees without *D. citri* feeding (Fig. 3). After 150 d, a similar pattern of *NPR1* and *PR-1* expression was observed between control trees without *D. citri* and trees exposed to continuous feeding.

### Temporal pattern of SA analytes in mature leaves of *Citrus sinensis*

**Fig. 4:**
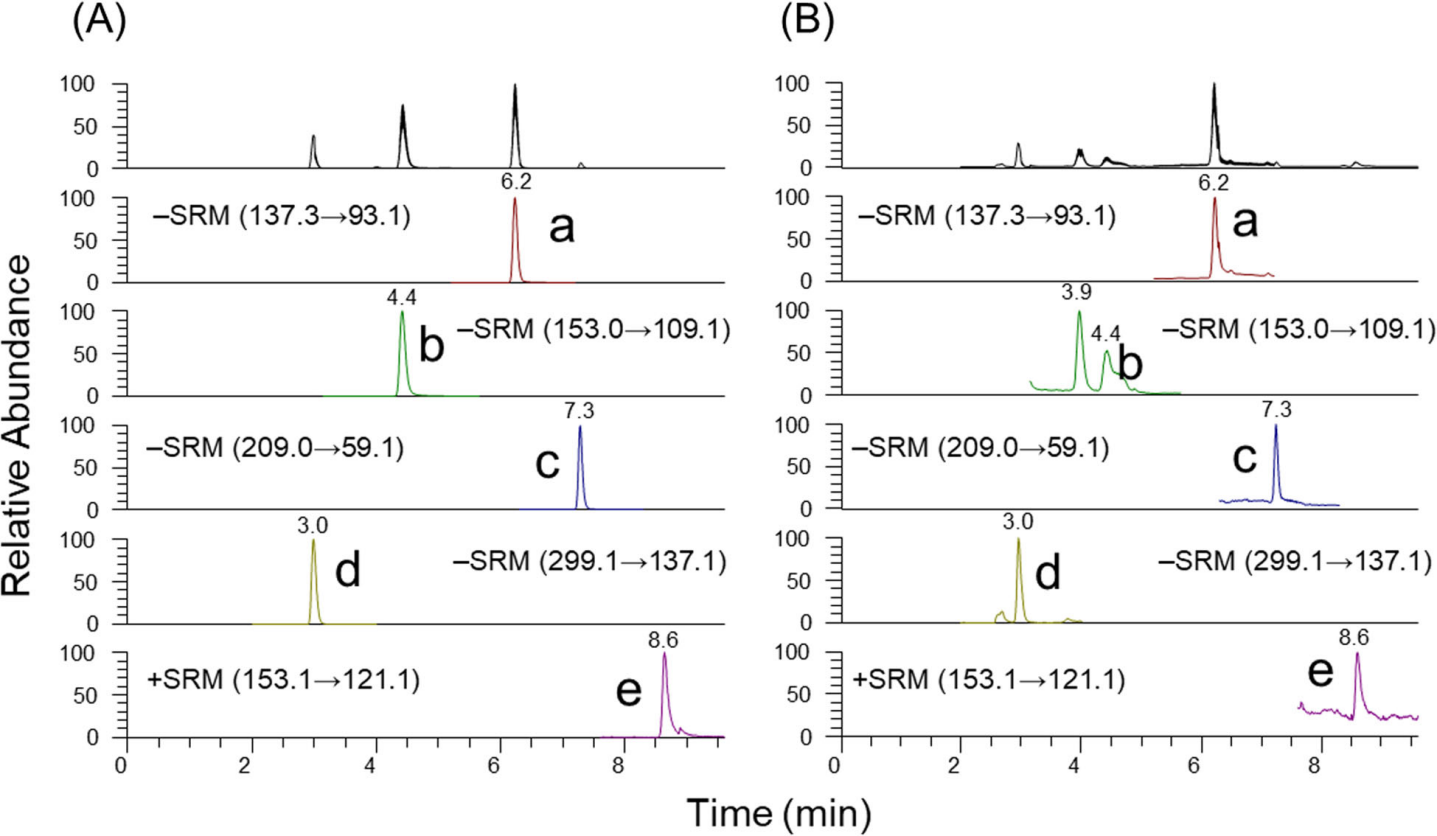
SRM chromatograms of (**a**) standards and (**b**) sample extracts: a. salicylic acid (SA), b. 2,3-dihydroxybenzoic acid (2,3-DHBA), c. jasmonic acid (JA), d. salicylic acid 2-O-β-D-glucoside (SAG), and e. methyl salicylate (MeSA)

**Fig. 5:**
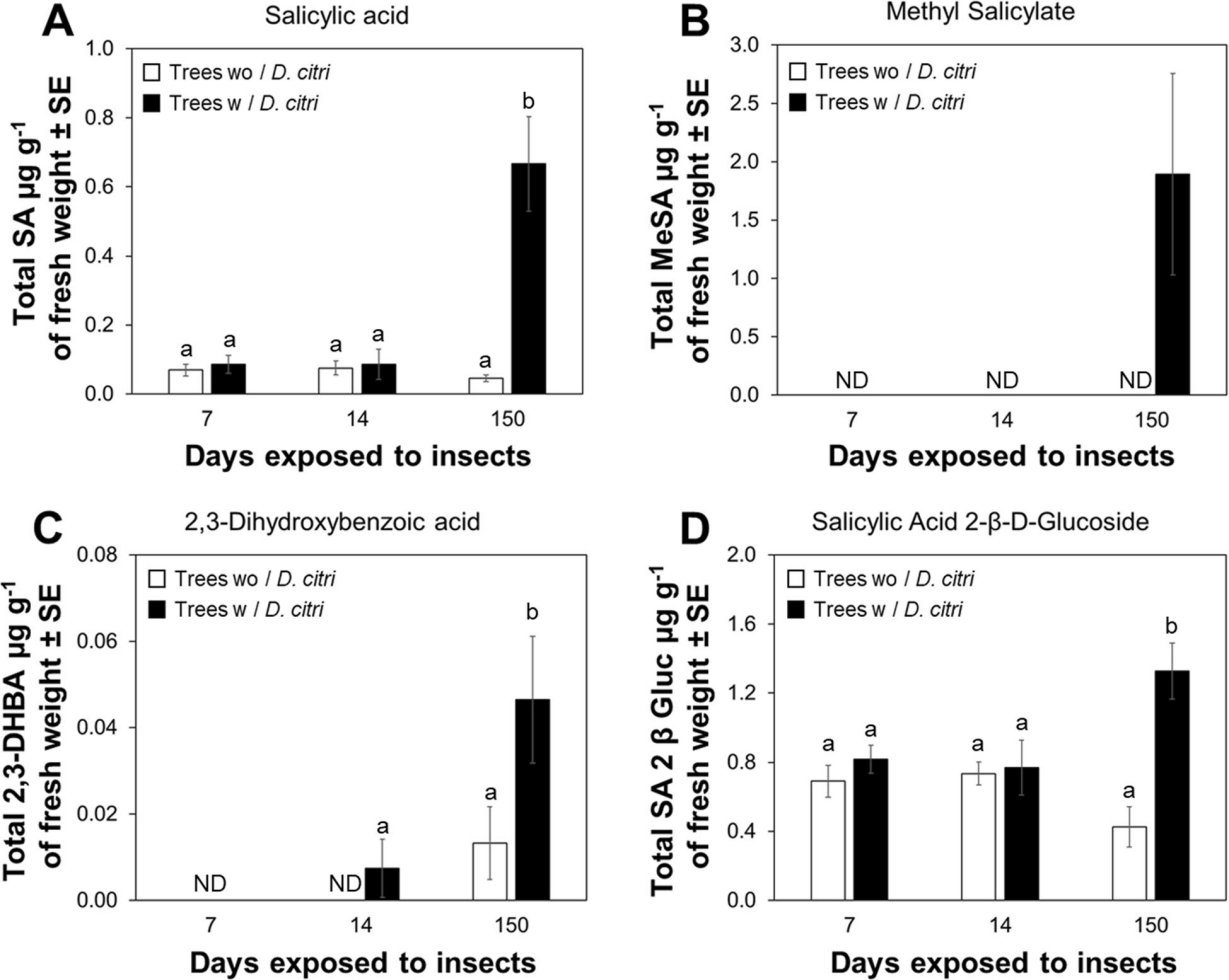
Feeding-induced expression pattern of SA metabolites in mature leaves of *Citrus sinensis*. **a** Quantification of total SA. **b** Quantification of methyl salicylate. **c** Quantification of 2,3-DHBA. **d** Quantification of salicylic acid 2-β-D-Glucoside. Data represent means ± standard error (SE) standard deviation (SD) of six biological replicates. The abbreviation ‘ND’ stand for Not detected. Different letters indicate statistical differences between treatments at *P* < 0.05 using one-way ANOVA with Tukey’s post hoc test

Selected reaction monitoring (SRM) chromatograms and spectra of standards and sample extracts are shown in Fig. 4 and Additional file 9: Figure S9, respectively. Quantification of SA and its metabolites using LC–MS analysis determined that accumulation of total SA in mature leaves at 7 and 14 d was not statistically different (*P* > 0.05) between treatments. However, SA accumulation increased significantly (*P* < 0.05) in plants exposed to continuous *D. citri* feeding for 150 d compared with trees without *D. citri* feeding (Fig. 5a).

The metabolite, MeSA, was only detected in mature leaves of plants exposed to 150 d of continuous *D. citri* feeding; it was not detected among trees without *D. citri* (Fig. 5b). The accumulation of 2,3-DHBA increased significantly in mature leaves of plants exposed to 150 d of continuous feeding by *D. citri* compared to the other durations of exposure or plants not exposed to *D. citri* (Fig. 5c). The glycosylated form of SA, salicylic acid 2-β-D-Glucoside (SAG), was observed at all time-points and its accumulation increased significantly (*P* < 0.05) in mature leaves of plants exposed for 150 d of continuous *D. citri* feeding compared to control plants (Fig. 4d). Overall, accumulation of SA and its metabolites increased significantly in plants exposed to 150 d of continuous feeding by *D. citri* (Fig. 5).

**Fig. 6:**
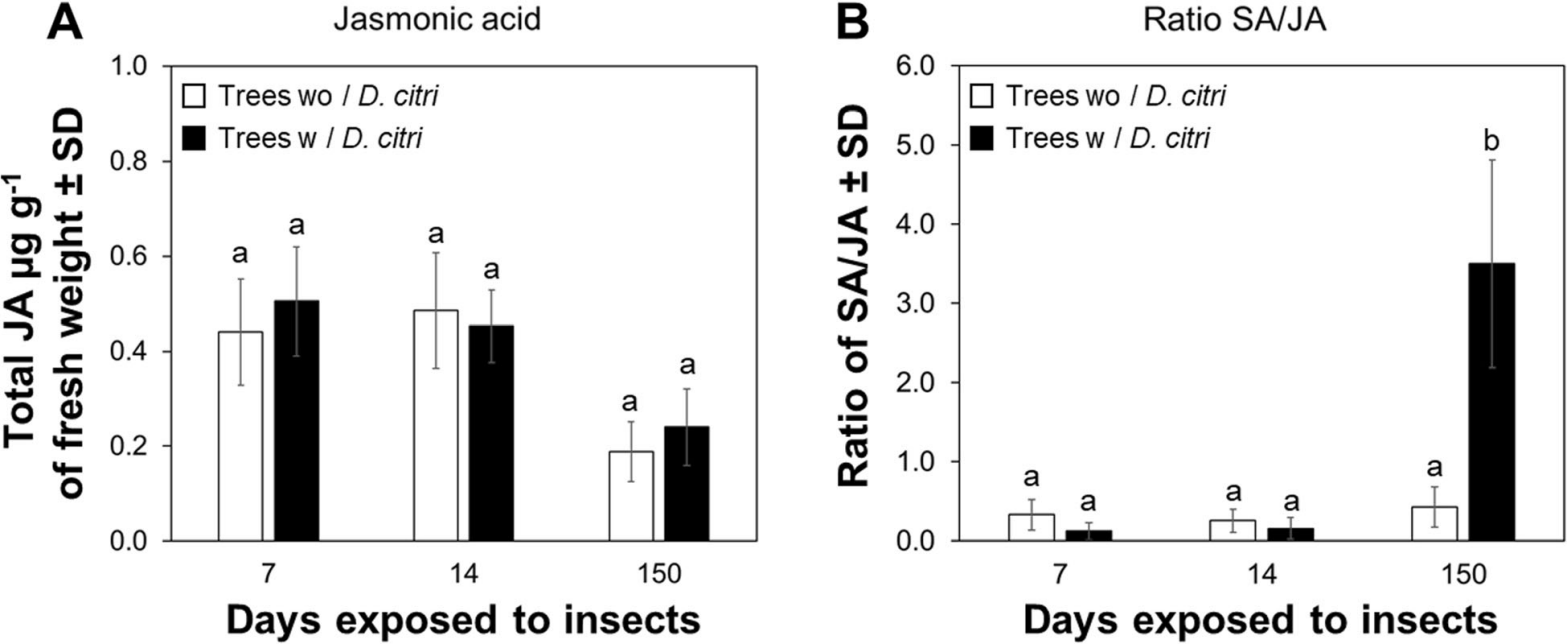
Amount of jasmonic acid and SA/JA ratio in mature leaves of *Citrus sinensis* in response to feeding by *Diaphorina citri*. **a** Quantification of jasmonic acid. **b** Ratio between salicylic and jasmonic acids. Data represent mean ± standard deviation (SD) of six biological replicates. Different letters indicate statistical differences between treatments at *P* < 0.05 were determined using one-way ANOVA with Tukey’s post hoc test

No statistical differences were observed in the accumulation of jasmonic acid (JA) in plants exposed to continuous feeding by *D. citri* compared to plants without insects (Fig. 6a). A well known increase in the ratio SA/JA equal to 3.50 was determined in leaves of plants exposed to 150 d of *D. citri* feeding (Fig. 6b).

## Discussion

Expression of genes involved in SA biosynthesis and associated with SA accumulation is known to increase in mixed age (juvenile-, moderate- and mature) *C. sinensis* leaves following 1 month of *D. citri* infestation [39]. Despite upstream regulation, the active SA level is also modulated by downstream metabolic modifications, including glycosylation, methylation, amino acid conjugation, and hydroxylation [16, 40]. These subsequent modifications are believed to serve different biological functions in plant defense [41]. It has been suggested that plants recognize phloem-feeding insects, such as aphids, whiteflies and psyllids as pathogens, activating an SA-dependent pathway response [8, 42]. Therefore, understanding the specific type of modulation occurring during SA metabolism and determining how it is associated with an SA-dependent pathway during *D. citri* feeding (infestation) may reveal important targets to disrupt the *C. sinensis - D. citri* interaction for disease management.

Proteins capable of catalyzing SA modifications have been described in detail by D’Maris Amick Dempsey, Vlot [16] and Maruri-López, Aviles-Baltazar [40]. In the current investigation, four genes (transcripts) involved in SA metabolism, and two involved in the SA-dependent pathway were identified from the *C. sinensis* genome and their expression pattern was analyzed at several time-points following initiation of continuous psyllid feeding on plant leaves. Candidate proteins involved in SA metabolism showed high similarities with their homologs in other plant species (Additional file 5: Figure S5, Additional file 6: Figure S6, Additional file 7: Figure S7 and Additional file 8: Figure S8), implicating these putative enzymes in the formation and hydrolysis of the various SA metabolites (MeSA, 2,3-DHBA, and SAG) measured here in *C. sinensis* (Fig. 5).

Methylation of SA is controlled by BA/SA carboxyl methyltransferase 1 (BSMT1), catalyzing the formation of the SA methyl ester, MeSA [18]. We observed a significant upregulation of *BSMT-like* (Fig. 2a) and concurrent downregulation of a methylesterase, *MES1-like* (Fig. 2b) in plants that had been exposed to *D. citri* feeding for 150 d. In Arabidopsis, a *AtBSMT1* mutant was unable to induce MeSA synthesis after *Pseudomonas syringae* infection, while overexpression of *AtBSMT1* caused accumulation of MeSA at the infection zone and these plants were unable to activate systemic acquired resistance (SAR) [43]. This result suggested that excessive levels of AtBSMT1 present in plant tissues outcompeted the activity or abundance of methylesterases (Sabp2 and MES). Similarly, an increase in MeSA production (Fig. 5b) associated with upregulation *BSMT-like* and concurrent downregulation of *MES1-like* documented here, suggest that prolonged feeding by *D. citri* on *C. sinensis* may inhibit SAR.

In Arabidopsis, hydroxylation of SA is controlled by salicylic acid 3 hydrolase (S3H), recently annotated in citrus as 2-oxoglutarate (2OG)/Fe (II)-dependent oxygenase (DMR6-like oxygenase). This protein was characterized by Zhang, Halitschke [25], and is known to synthetize hydroxylated SA metabolites: 2,3-DHBA and 2,5-DHBA. Citrus plants infested with *D. citri* for 150 d exhibited an upregulation of *DMR6-like* (Fig. 2c); this upregulation was consistent with an increased concentration of 2,3-DHBA in mature leaves (Fig. 5c). In Arabidopsis, overexpression of *DMR6-like oxygenase* increased plant susceptibility to the fungus, *Hyaloperonospora arabidopsidis*, and induced the development of disease-associated chlorosis [44]. Another investigation showed that expression of *DMR6-like oxygenase* in senescing leaves of *A. thaliana* was upregulated by elevated endogenous levels of SA in leaves [25]. Similarly, our analysis determined analagous regulation (high levels of SA associated with increased expression of *DMR6-like oxygenase*) in leaves of *C. sinensis* exposed to prolonged *D. citri* feeding (Fig. 2c).

The transformation of SA into SAG occurs in the cytoplasm of soybean and tobacco [45, 46]. In Arabidopsis, two UDP-dependent glycosyltransferases (UGT74F1 and UGT74F2) have been described with differences in activities forming either SAG or SA glucose ester [22, 24]. Song, Koo [47] determined that overexpression of UGT74F2 (also annotated as AtSGT1) results in lower levels of SA leading to increased plant susceptibility to *P. syringae*. An objective of our investigation was to determine whether *D. citri* could modulate expression of *UGT74F2* and SAG levels. Our results indicated a decrease in *UGT74F2* expression (Fig. 2d) with increased levels of SAG (Fig. 5a, d) in intact leaves of citrus plants following prolonged exposure to *D. citri* feeding and this did not occur in comparable plants without insects. Therefore, an analogous regulation between gene expression and SAG levels in *C. sinensis* was not observed, suggesting that SAG accumulation levels in citrus are likely regulated by: i) another enzyme with higher specificity for SAG formation, or ii) *UGT74F2* transcript expression reached a maximum level prior to the time course selected for analysis in this study.

The overaccumulation of SA observed in leaves of citrus plants following prolonged exposure to *D. citri* feeding may be related with plant growth and an SA-dependent pathway. Despite the importance of SA as a plant hormone regulating several plant biological processes, its role in plant growth has been not been investigated in detail, and is mostly confined to Arabidopsis as a plant model. Arabidopsis mutants created to constitutively express high levels of SA display a ‘dwarf phenotype’ with a decreased growth rate in both above- and below-ground tissues, which in some cases can result in plant death [48, 49]. In our investigation, vegetative growth (above-ground) of *C. sinensis* following prolonged *D. citri* infestation was stunted or possessed less canopy (Additional file 10: Figure S10), suggesting that high levels of SA quantified in leaves were associated with reduced growth/canopy size observed with *D. citri*-infested trees. Also, high SA concentration is associated with NPR1 degradation in Arabidopsis [50]. It is possible that the lack of *PR-1* upregulation in *D. citri*-infested trees (Fig. 3b) is caused by an imbalance in NPR1 homeostasis, and further investigation of proteins associated with regulating NPR1 stability and activity, such as the NPR1 paralogues NPR3 and NPR4, is needed to fully understand this SA-dependent pathway.

## Conclusions

**Fig. 7:**
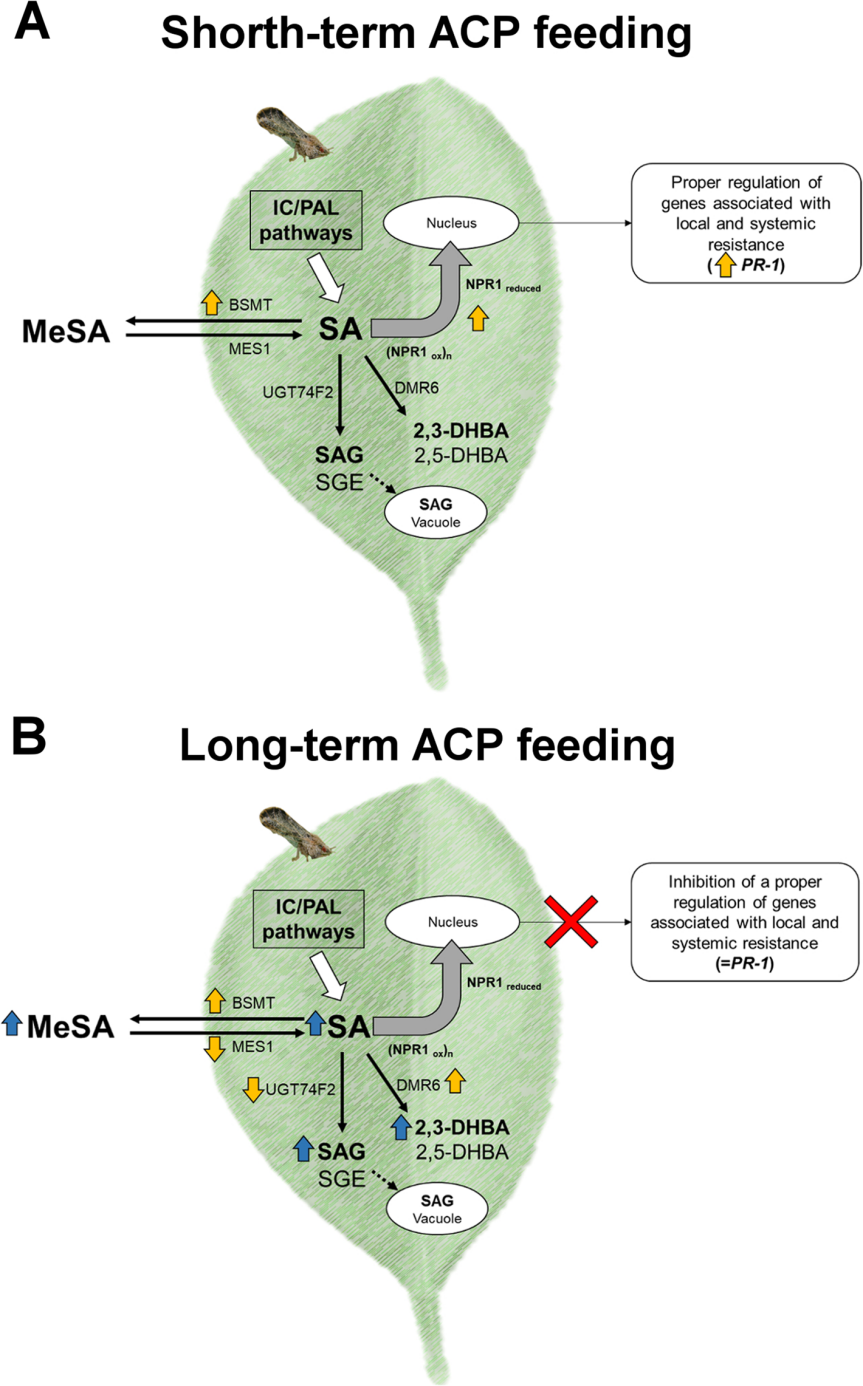
Models associated with *Diaphorina citri* feeding and plant immune response in leaves of *Citrus sinensis*. Hypothetical molecular mechanisms involved in transcriptional regulation of SA modification and activation of defense-associated responses via NPR1 in *C. sinensis*. **a** Model of plant immune response to short-term *D. citri* feeding. **b** Model of plant immune response to long-term *D. citri* feeding. Abbreviations are similar to Fig. 1; black line arrows indicate synthesis of SA metabolites. Dashed lines indicate transport to vacuole; thick, white arrows indicate the Isochorismate (IC) Phenylalanine ammonia-lyase pathways; thick, gray arrows indicate the reduction and monomerization of NPR1; thick, orange arrows indicate upregulation of genes and thick, blue arrows indicate the increase in SA and its metabolites in leaves. (=*PR-1*) represents no changes in transcriptional regulation

We described the molecular mechanisms involved in: i) transcriptional regulation of SA modification, and ii) activation of defense-associated responses in leaves of *C. sinensis* challenged by various durations of *D. citri* feeding injury. We propose two scenarios that describe transcriptional regulation of SA modification and activation of defense-associated responses via NPR1 in *C. sinensis* depending on duration of plant exposure to insect injury (Fig. 6a, b). Following short-term exposure to *D. citri* feeding, the immune response of citrus was upregulated with increased expression of NPR1 and *PR-1* and there was no associated accumulation of SA. However, prolonged feeding by *D. citri* altered the transcription of several genes (Fig. 7b), resulting in excessive accumulation of SA and its metabolites (Fig. 5) in mature leaves. We postulate that transcriptional regulation of SA-related genes in citrus following prolonged *D. citri* feeding disrupts homeostasis of the SA pathway compromising local and systemic acquired resistance. Therefore, feeding injury caused by the vector may facilitate disease progression unrelated to *Candidatus* Liberibacter asiaticus (CLas) pathogenesis.

Within the context of HLB management, our results suggest that injury associated to a long-term *D. citri* feeding may significantly compromise plant growth in addition to pathogen-related decline, such as phloem plugging in *C*Las-infected citrus trees [51]. These results further support vector suppression in areas where HLB disease is endemic as a short-term management strategy. However, long-term solutions such as insect and/or disease tolerant varieties and/or therapies targeting physiologically expressed insect injury or pathogenesis-related symptoms based on transient gene expression are ultimately necessary.

## Methods

### Citrus husbandry

All of the plant materials were produced and purchased from commercial Southern Citrus Nurseries, Dundee, Florida in August of 2017. Plants used in experiments were 2-yr old uninfected [*Candidatus* Liberibacter asiaticus (CLas)-free] *Citrus sinensis* L. Osbeck cv Valencia grafted onto US-812 rootstocks [52], After 2 months, citrus plants were transplanted into new plastic pots (19.69 × 45.7 cm) and filled with a Fafard citrus RSI soil mix containing Canadian sphagnum peat moss, perlite, vermiculite, dolomitic limestone, RSI and Pluronic (Sungro, Horticulture Distribution, Inc., WA, USA). Plants were repositioned into growth chambers and maintained under the following conditions: 23 ± 3 °C, 60RH, and a 16:8 h (Light: Dark) photoperiod with a maximum photosynthetic radiation of 215 μmol s^− 1^ m^− 2^. Plants were watered twice per week, and fertilized twice per month with an alternating schedule of a 24–8-16 NPK solution at 4 g L^− 1^ (Miracle-Gro All Purpose Plant Food; Scotts Miracle-Gro Products, Marysville, OH) and a 6–4-6 (N–P–K) granular fertilizer at 1 g per pot (Expert gardener Gro Tec. Inc. Madison, GA).

### Insect rearing

The CLas-free *D. citri* used in this study were reared on 3–4 yr. old *Citrus sinensis* cv Valencia trees maintained in a greenhouse at 26 ± 2 °C, 60–65% RH, and a 16:8 h (Light: Dark) photoperiod. Before initiating experiments, the presence/absence of CLas was examined in at least 40 *D. citri* individuals to confirm the lack of infection using TaqMan qPCR.

### DNA extraction and CLas detection by TaqMan qPCR assay

Genomic DNA from single insects was isolated using the DNeasy blood and tissue kit (Qiagen Inc., Valencia, CA), following the manufacturer’s protocol. Quantity and purity of DNA samples were measured on a Nanodrop 2000 Spectrophotometer (Thermo fisher Scientific, Waltham, MA). Genomic DNA of CLas was detected using previously described target probes for CLas-specific 16S rDNA and an internal control sequence (gene region) for *D. citri* (*Wingless*) [53]. DNA amplifications were conducted in ABI 7500 qPCR system (Applied Biosystems, Foster City, CA) using 96-well MicroAmp reaction plates (Applied Biosystems). Each TaqMan qPCR reaction was achieved using 100 ng of genomic DNA, 100 nM of each dual-specific labeled probe-primer sets, TaqMan® Universal PCR Master Mix (Applied Biosystems) and adjusted to a final volume of 20 μL with molecular grade pure water. The conditions used for TaqMan assays consisted of: an incubation at 50 °C for 2 min, a polymerase activation step at 95 °C for 10 min, followed by 40 cycles at 95 °C for 15 s and 60 °C for 60 s. Each 96-well plate contained samples including a ‘no template’ control, a positive control (CLas-infected samples), negative control (CLas-uninfected samples). Reactions were performed in duplicates and considered positive for target sequences if the cycle quantification (Cq) value, determined by the ABI 7500 Real-Time software (version 1.4, Applied Biosystems), was ≤36 cycles.

### Bioinformatic analyses

#### Selection of genes

The expression pattern of genes involved in SA modification and SA-dependent defense signaling in *C. sinensis* L. Osbeck cv Valencia was investigated with a search for homologs in *A. thaliana.* The Blast algorithm was used to select genes according to percentage of identity with *A. thaliana* homologous. We selected *Methylesterase 1* (*MES1*, Accession number NM_127926), *2-oxoglutarate (2OG)/Fe(II)-dependent oxygenase* (Accession number NM_117118) [previously annotated and described by Zhang, Halitschke [25] as *salicylic acid 3-hydroxylase* (*S3H or DMR6*)]*, S-adenosyl-L-methionine-dependent methyltransferase* (*BSMT1*, Accession number NM_111981), *UDP-glucosyltransferase 74F2* (*UGT74F2*, Accession number NM_129944), *Non-expressor of Pathogenesis Related genes 1* (*NPR1*, Accession number NM_105102), and *Pathogenesis-related protein 1* (*PR-1*, Accession number NM_127025). After gene-selection, RT-qPCR primers for each candidate transcript were designed *in Primer3 web* [54] and phylogenetic analyses were performed for each protein family.

#### Phylogenetic analyses of genes associated to SA modifications

The open reading frame of each *C. sinensis* gene chosen was aligned with a homologous nucleotide sequences obtained from *A. thaliana*, *Arabidopsis lyrata*, and Citrus clementina. A multiple nucleotide sequence alignment was performed using ClustalW in the Cyberinfrastructure for Phylogenetic Research (CIPRES) portal [55]. The estimation of phylogeny was achieved using MrBayes 3.2.6 in the CIPRES portal, with the following settings: four chains, two runs, nucleotide model = Generalized time reversible (GTR), rate variation = “invgamma” (GTR + I + Gamma model). Analysis of the Metropolis-coupled Markov chain Monte Carlo (MCMC) was run for fifteen million generations, sampled every 5000th step, and the first 25% of sampled trees were discarded as burn-in. The values of branch support were obtained by the method of posterior probability (≥0.70). Each phylogenetic tree was rooted at midpoint and edited in the Figtree program v.1.4.0 [56].

#### Alignments

Before creating amino acid alignments, only the genes that showed a unique amplicon were selected for amino acid alignments. For each transcript associated with SA modifications and signaling, the Open Reading Frame (ORF) was obtained using the Open Reading Frame Finder tool (http://www.ncbi.nlm.nih.gov/gorf/orfig.cgi*)*. The sequences used for DMR6-like oxygenase *alignment were: C. sinensis* (accession number KK784903), *A. thaliana* (accession number NM_117118), *Nicotiana tabacum* (accession number NM_001325946), and *Zea mays* (accession number NCVQ01000003). The sequences used for S-adenosyl-L-methionine-dependent methyltransferase superfamily protein (*BSMT*) alignment were*: C. sinensis* (accession number XM_006466773), *A. thaliana* (accession number NM_111981), *N. tabacum* (accession number NM_001324786), and *Z. mays* (accession number CM000785). The sequences used for Methylesterase alignment were*: C. sinensis* (accession number XM_015532488), *A. thaliana* (accession number NM_127926), *N. tabacum* (accession number NM_001325513), and *Z. mays* (accession number XM_008676727). Finally, the sequences used for UDP-glycosyltransferase 74F2 alignment were: *C. sinensis* (accession number XM_006478492), *A. thaliana* (accession number NM_129944), *N. tabacum* (accession number XM_016625845), and *Z. mays* (accession number NCVQ01000003). All alignments were performed using ClustalW in Bioedit version 7.2.5 [57]. To analyze the percentage of identity and similarity between the amino acid sequences of *C. sinensis* and *A. thaliana*, pairwise alignments were performed using the Blast2 sequences algorithm [58]. The predicted conserved domains in each amino acid sequence were determined using the NCBI Conserved Domain Database search [59]*.*

### Insect feeding and plant-tissue collection

The objective of this experiment was to compare gene expression and SA metabolites produced during SA conversion between trees exposed to a constantly reproducing population of uninfected *D. citri* (feeding treatment) versus trees without insects (control treatment). There was no CLas infection present throughout the experiment in either vector or host. The feeding treatment consisted of initial release of 20 uninfected *D. citri* (~ 50:50 male: female ratio) per plant that were then constantly exposed to a reproducing population of *D. citri* adults and nymphs feeding on plants. Control plants were handled identically but remained unexposed to psyllids. The experiment began during spring flush (April 2018) when six plants of similar size and phenology were individually housed within (58.4 × 58.4 × 88.9 cm) insect-proof cages per treatment. From each plant, 19.63 mm^2^ leaf discs were collected from the midribs of 9 mature leaves following various durations of insect feeding to observe plant response after short (7-, 14- d of exposure to insects) and long-term (150- d exposure to insects) feeding by *D. citri*. The ‘150 d’ time point was chosen because plants began to display decreased growth rate compared to plants without insects (Additional file 10: Figure S10) near this time point. Plant tissues were immediately flash frozen using liquid nitrogen, ground in a tissue lyser, and then stored at − 80 °C until further analyses.

### RNA extraction and cDNA synthesis

The total RNA was extracted from 20 mg of previously ground plant tissue using the RNeasy Plant mini kit (Qiagen) following the manufacturer’s instructions. To remove genomic DNA, each sample was treated with DNase using the Turbo DNase kit (Ambion) following the manufacturer’s protocol. The quantity and purity of total RNA was determined in a Nanodrop 2000 Spectrophotometer (Thermo fisher Scientific, Waltham, MA). After DNA removal, synthesis of complementary DNA (cDNA) was performed. Each cDNA synthesis reaction consisted of 500 ng of total RNA, 5X cDNA synthesis buffer, anchored-Oligo (dT) primers, RT Enhancer and Verso Enzyme Mix, carried out with the Verso cDNA Synthesis kit (Thermo Fisher scientific, CA), as per the manufacturer’s protocol. Thereafter, cDNA samples were stored at − 20 °C until further analyses.

### Gene expression analyses by RT-qPCR

RT-qPCR reactions were performed using PowerUp™ SYBR® Green Master Mix (ThermoFisher scientific). Each qPCR reaction contained 10 ng of cDNA (template), 300 nM of each gene-specific primer (Table 1), and 1x of PowerUp™ SYBR® Green Master Mix; the final volume was adjusted with nuclease-free water to 20 μL. The real-time PCR program consisted of a UDG activation step at 50 °C for 2 min, a denaturation step at 95 °C for 2 min, followed by 40 cycles at 95 °C for 5 s and 60 °C for 60 s. Real-time PCRs were performed using an Applied Biosystems 7500 Real-Time PCR System (Thermo Fisher Scientific). Each RT-qPCR reaction was performed in duplicate with a negative control in each run. Primer specificity was monitored with melting curve analysis using QuantStudio™ software V1.3 (Thermo Fisher Scientific) and 2% agarose gel electrophoresis. The relative gene expression for each target gene was determined with the delta delta CT (*ΔΔCT*) method [60] using the mean CT of β-actin and Elongation factor-1α (Accession numbers XM_026823249.1 and XM_006488084, respectively*)* as reference genes [61], following the equation: *ΔΔCT = (CT*_*, Target*_ *– CT*_*, Actin/EF1a*_*)*_*Time x*_ *- (CT*_*, Target*_ *– CT*_*, Actin/EF1a*_*)*_*Time 0*_, where ‘time x’ corresponded to the ΔCT values observed between the target gene and the mean CT of β-actin and Elongation factor-1α following specified durations (7, 14 and 150 days) of plant exposure to treatments, while ‘time 0’ corresponded to the ΔCT values of genes analyzed in plants without insects, 1 day before beginning the assays.

**Table 1:** Primers used to investigate effects of feeding by *Diaphorina citri* on transcriptional regulation of genes involved in SA-dependent defense signaling pathway and modification of SA

Primers		
Purpose	Name	Sequence
Expression analysis	*Cs BSMT-like* F	5-GGCTCTCAACAATATGGTCTCC-3
	*Cs BSMT-like* R	5-TTTATTTCAGCTGGTGATGGTG-3
	*Cs UGT74F2-like* F	5-AGGGTCAAAGTGGGAGAGAAA-3
	*Cs UGT74F2-like* R	5-TATCTGAAGTGCCACCCTCAC-3
	*Cs DMR6-like* F	5-TCTTCCAGGTGAGGAACCAC-3
	*Cs DMR6-like* R	5-CTGGCGATTTGAAAAATGCT-3
	*Cs MES1-like* F	5-AGACACCAAACACCAACCATCT-3
	*Cs NPR1* F	5-TGATAAGACCTTGCCACAACAC-3
	*Cs NPR1* R	5-ACCGCAGGATTCAGATCTATGT-3
	*Cs PR-1* F	5-ACTGCAATCTTGTGCATTCG-3
	*Cs PR-1* R	5-TTCACCCACAGTTTCACAGC-3
	*Cs MES1-like* R	5-CTGAAAATTGAGTGTCCAACCA-3
	*Cs Actin* F	5-AGCCTACATTGCGCTTGACT-3
	*Cs Actin* R	5-ATGACCTGCCCATCAGGTAG-3
	*Cs Elongation factor 1α* F	5-CCAAGGATGGTCAGACTCGT-3
	*Cs Elongation factor 1α* R	5-GCGTCCATCTTGTTACAGCA-3

### Metabolite analysis by liquid chromatography-mass spectrometry (LC-MS)

SA metabolites were extracted using 20 mg of ground leaves. Briefly, each sample was mixed with 0.25 mL of ice-cold methanol/water solution (20/80, v/v), including three internal standards (0.01 μg/mL salicylic acid-d_6_, for salicylic acid and jasmonic acid; 0.4 μg/mL 2,5-dihydroxybenzoic acid-d_3_, for salicylic acid 2-O-β-D-glucoside and 2,3-DHBA; and 0.8 μg/mL methyl salicylate-d_4_ for MeSA). Samples were extracted by ultra-sonic assisted extraction for 30 min in ice block-filled bathtub. After incubation, samples were centrifuged at 30,000 g at 4 °C during 10 min, the supernatant was recovered and filtered with a 0.22 μm membrane filter, and 10 μL was injected into LC–MS/MS system.

LC–MS/MS analyses were carried out with an Ultimate 3000 LC system coupled to a TSQ Quantiva triple quadrupole mass spectrometer (Thermo Fisher Scientific, San Jose, CA, USA). The analytes (salicylic acid 2-O-β-D-glucoside, 2,3-DHBA, salicylic acid, jasmonic acid, and methyl salicylate) were chromatographed on a Thermo Fisher scientific Acclaim C30 column (150 mm × 2.1 mm, 3.0 μm particle size) at a column temperature of 30 °C using a gradient elution with 0.1% formic acid in water (eluent A) and 0.1% formic acid in acetonitrile (eluent B). The gradient was as follows: 0–10 min 20–95% B and 10–15 min 95% B. The column was re-equilibrated using the initial mobile phase before next run. The flow rate was set at 0.2 mL/min. The mass spectrometer was operated in both positive and negative electrospray ionization (ESI+ and ESI–) with selected reaction monitoring (SRM) mode. The ESI parameters were as follows: spray voltage, 3500 V for ESI+ and 2500 V for ESI–; sheath gas, 35 Arb; aux gas, 10 Arb; ion transfer tube temperature, 325 °C; and vaporizer temperature, 275 °C. Dwell time was 100 msec, and collision-induced dissociation (CID) gas was 2 mTorr. The parameters of MS/MS (retention time, SRM transition, collision energy and RF lens) are presented in Table 2. The analytes were assigned by comparing SRM transitions and retention times with authentic standards. The absolute quantification was performed using calibration curves constructed by plotting peak area ratios of the analyte relative to the internal standard against analyte concentrations (Table 3). Pooled quality control (QC) samples were run after every six experimental samples to check the batch reliability. Xcalibur software (Ver. 3.0) was employed for data processing and instrument control.

**Table 2:** Optimal MS/MS parameters including SRM transition, collision energy and RF lens for target metabolites

Analyte	Retention time (min)	Polarity	Q1 (m/z)	Q3 (m/z)	CE (V)	RF lens (V)
Salicylic acid 2-O-β-D-glucoside	3.0	Negative	299.1	137.1	15	68
2,3-Dihydroxybenzoic acid	4.4	Negative	153.0	109.1	16	51
2,5-Dihydroxybenzoic acid -d_3_ (IS)	4.0	Negative	156.0	111.1	23	57
Salicylic acid	6.2	Negative	137.3	93.1	17	50
Jasmonic acid	7.3	Negative	209.0	59.1	13	48
Salicylic acid -d_6_ (IS)	6.2	Negative	141.3	97.1	17	45
Methyl salicylate	8.6	Positive	153.1	121.1	15	43
Methyl salicylate-d_4_ (IS)	8.6	Positive	157.1	125.1	15	50

*Abbreviation*: *IS* correspond to Internal standard

**Table 3:** Linearity for target metabolites produced by calibration curves

Analyte	Linear range (ng/mL)	Regression equation	r2
Salicylic acid 2-O-β-D-glucoside	0.8–40	y = 0.01237x + 0.00013	0.9989
2,3-Dihydroxybenzoic acid	0.8–40	y = 0.04061x + 0.00078	0.9992
Salicylic acid	0.8–40	y = 0.09231x - 0.00041	0.9995
Jasmonic acid	0.8–40	y = 0.00238x - 0.00010	0.9985
Methyl salicylate	8–400	y = 0.00157x - 0.00014	0.9987

### Statistical analyses

The relative gene expression and quantity of SA metabolites were analyzed using analysis of variance (ANOVA) with Tukey’s test post hoc analyses. All statistical analyses were performed using RStudio environment [62].

## Supplementary information

**Additional file 1:** **Figure S1.** Phylogenetic analysis of Salicylate/benzoate carboxyl methyltransferases (BSMT). Bayesian analysis was performed using the ORF of *BSMT-like* from *C. sinensis* and compared to homologous proteins from *A. thaliana, A. lyrata* and *C. clementina*. Numbers at the nodes denote posterior probabilities. The reference bar indicates the distance (number of amino acid substitutions per site).

**Additional file 2:** **Figure S2.** Phylogenetic analysis of DMR6-like oxygenases (DMR6). Bayesian analysis was performed using the ORF of *DMR6-like* from *C. sinensis* and compared to homologous proteins from *A. thaliana, A. lyrata* and *C. clementina*. Numbers at the nodes denote posterior probabilities. The reference bar indicates the distance (number of amino acid substitutions per site).

**Additional file 3 :** **Figure S3.** Phylogenetic analysis of Methylesterases (MES). Bayesian analysis was performed using the ORF of *MES1-like* from *C. sinensis* and compared to homologous proteins from *A. thaliana, A. lyrata* and *C. clementina*. Numbers at the nodes denote posterior probabilities. The reference bar indicates the distance (number of amino acid substitutions per site).

**Additional file 4:** **Figure S4.** Phylogenetic analysis of UDP-glycosyltransferases 74 (UGT74). Bayesian analysis was performed using the ORF of *UGT74F2-like* from *C. sinensis* compared to and homologous proteins from *A. thaliana, A. lyrata* and *C. clementina*. Numbers at the nodes denote posterior probabilities. The reference bar indicates the distance (number of amino acid substitutions per site).

**Additional file 5: Figure S5.** Alignment of S-adenosyl-L-methionine-dependent methyltransferase. Salicylate carboxymethyltransferase from *C. sinensis* was aligned with one species from each plant order (*Solanales, Brassicales and Poales). Solanales represented by Nicotiana tabacum, Brassicales by Arabidopsis thaliana*, and Poales by *Zea mays*. The Methyltransf_7 domain (pfam03492) found within these sequences is indicated by an underlined round dot line.

**Additional file 6:** **Figure S6.** Amino acid alignment of DMR6-like oxygenase 1. DMR6-like oxygenase 1 from *C. sinensis* was aligned with one species from each plant order (*Solanales, Brassicales and Poales). Solanales represented by Nicotiana tabacum, Brassicales by Arabidopsis thaliana*, and Poales by *Zea mays*. The Oxidoreductase domain (PLN02912) found within these sequences was featured using an underline round dot line.

**Additional file 7: Figure S7.** Alignment of Methylesterases. Methylesterase1-like from *C. sinensis* was aligned with one species from each plant order (*Solanales, Brassicales and Poales). Solanales represented by Nicotiana tabacum, Brassicales by Arabidopsis thaliana*, and Poales by *Zea mays*. The Methyl indole-3-acetate methyltransferase domain (PLN02211) found within these sequences is indicated by underlined round dot line.

**Additional file 8:** **Figure S8.** Alignment of UDP-Glycosyltransferase 74F2. UGT74F2 from *C. sinensis* was aligned with one species from each plant order (*Solanales, Brassicales and Poales). Solanales represented by Nicotiana tabacum, Brassicales by Arabidopsis thaliana*, and Poales by *Zea mays*. The Glycosyltransferase family 1 domain (cl10013) found within these sequences is indicated by underlined round dot line.

**Additional file 9:** **Figure S9.** SRM spectra of (A) and (B) sample extract: a. salicylic acid (SA), b. 2,3-dihydroxybenzoic acid (2,3-DHBA), c. jasmonic acid (JA), d. salicylic acid 2-O-β-D-glucoside (SAG), and e. methyl salicylate (MeSA).

**Additional file 10:** **Figure S10.** Representative image of *C. sinensis* plants. Left panel shows representative control plants after 150 days. Right panel shows plants exposed to insect feeding for 150 days.

## Data Availability

Data and materials are available on request from the corresponding author. This study does not use large datasets consequently no supplementary data has been deposited in public repositories.

## Abbreviations

2,3-DHBA: 2,3-Dihydroxybenzoic acid
ACP: Asian citrus psyllid
BSMT: Benzoic acid/salicylic acid carboxyl methyltransferase
*C*Las: *Candidatus* Liberibacter asiaticus
HLB: Huanglongbing
MES: Methylesterase
MeSA: SA methyl ester (methyl salicylate)
NPR1: Nonexpresser of Pathogenesis Related genes 1
PR-1: Pathogenesis Related gene 1
SA: Salicylic acid
SAG: 2-O-β-D-glucoside
SAR: Systemic acquired resistance
UGT: UDP-glucosyltransferases
