# Supplementary figures and images for: Long-term, sustained feeding by Asian citrus psyllid disrupts salicylic acid homeostasis in sweet orange
Source: BMC Plant Biol. 2019 Nov 12;19:493. doi: 10.1186/s12870-019-2114-2 (PMC6852996; doi:10.1186/s12870-019-2114-2)

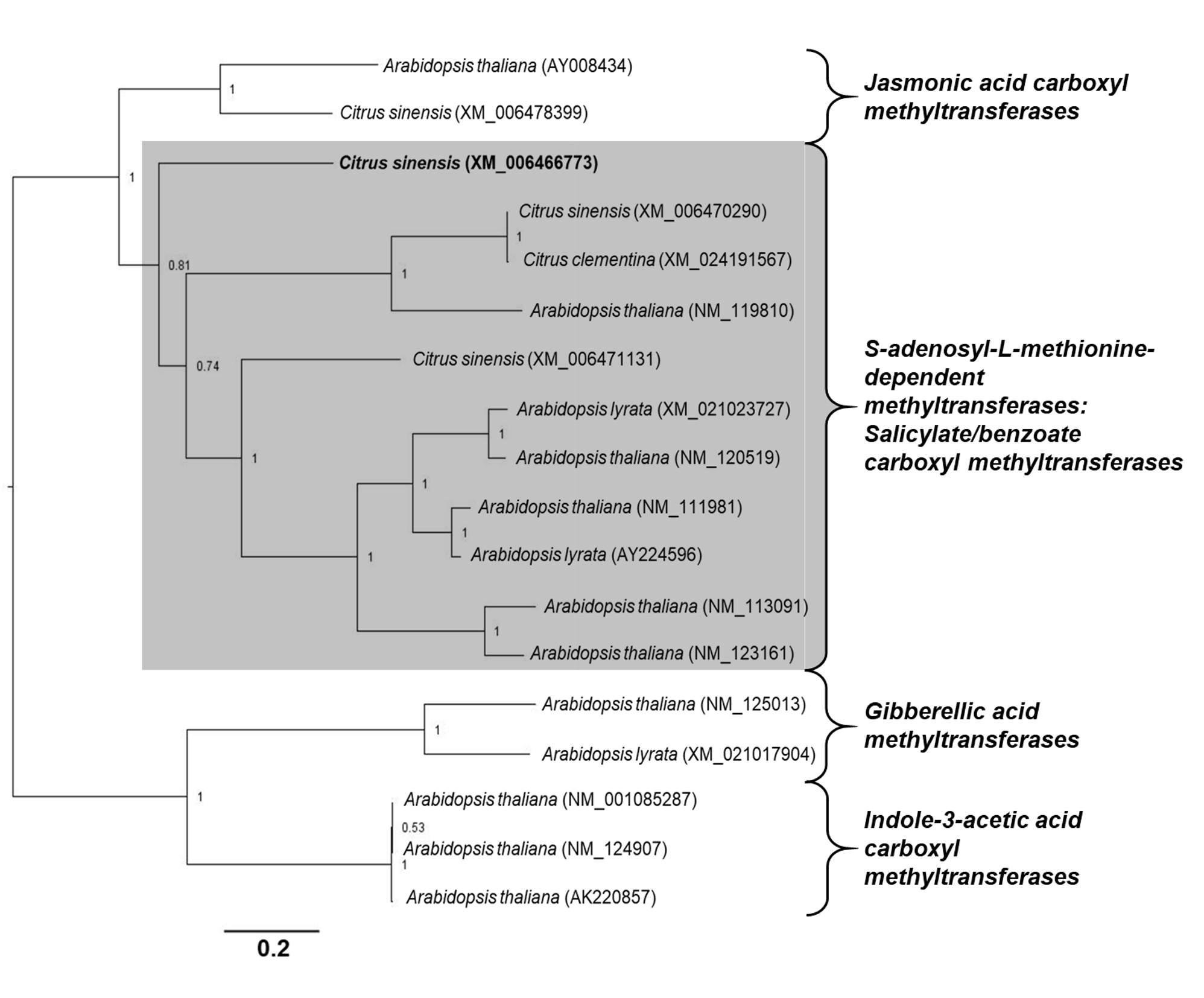

Supplement: Supplementary file 1 — Additional file 1: Figure S1. Phylogenetic analysis of Salicylate/benzoate carboxyl methyltransferases (BSMT). Bayesian analysis was performed using the ORF of BSMT-like from C. sinensis and compared to homologous proteins from A. thaliana, A. lyrata and C. clementina. Numbers at the nodes denote posterior probabilities. The reference bar indicates the distance (number of amino acid substitutions per site). [file 12870_2019_2114_MOESM1_ESM.jpg]

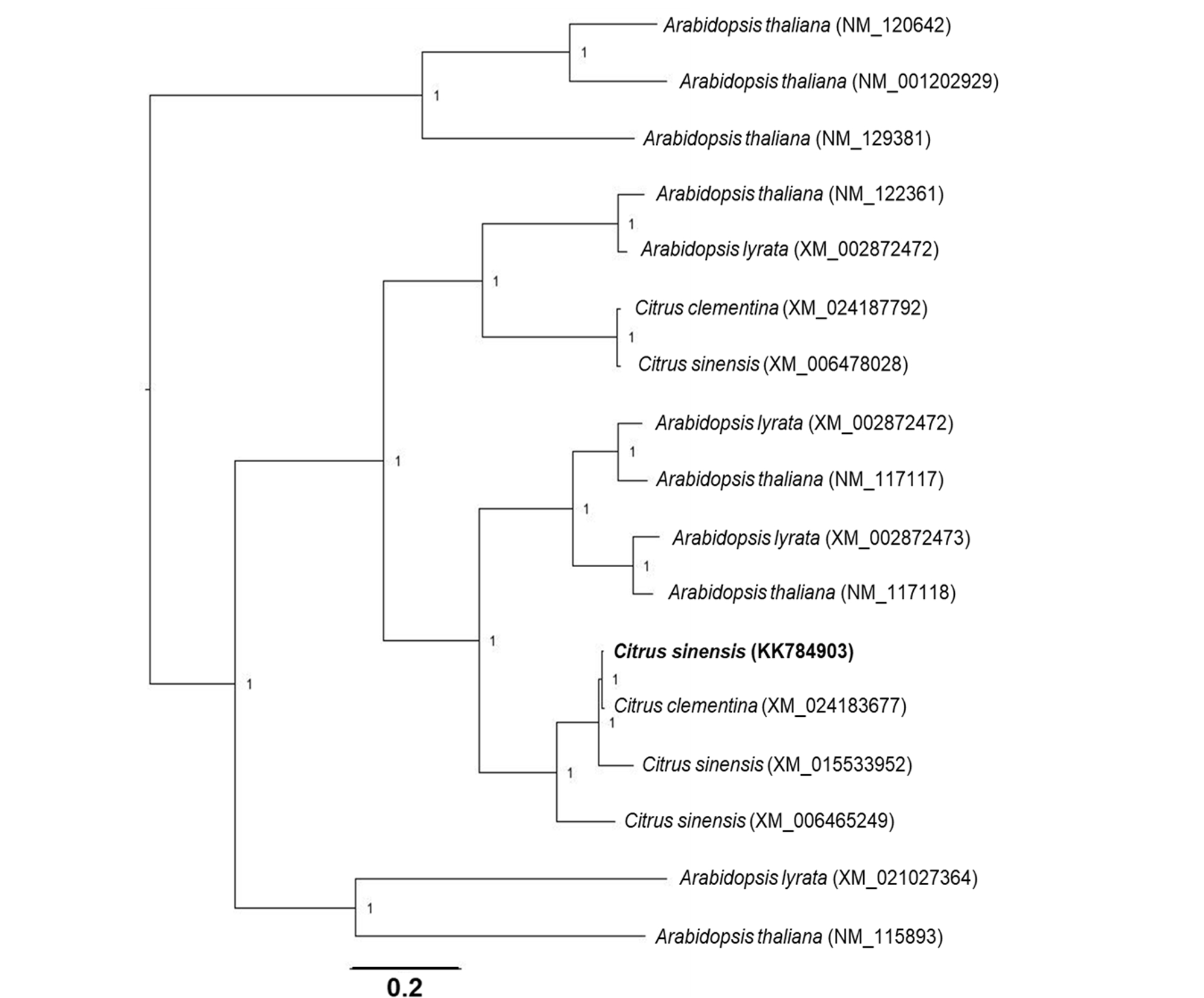

Supplement: Supplementary file 2 — Additional file 2: Figure S2. Phylogenetic analysis of DMR6-like oxygenases (DMR6). Bayesian analysis was performed using the ORF of DMR6-like from C. sinensis and compared to homologous proteins from A. thaliana, A. lyrata and C. clementina. Numbers at the nodes denote posterior probabilities. The reference bar indicates the distance (number of amino acid substitutions per site). [file 12870_2019_2114_MOESM2_ESM.jpg]

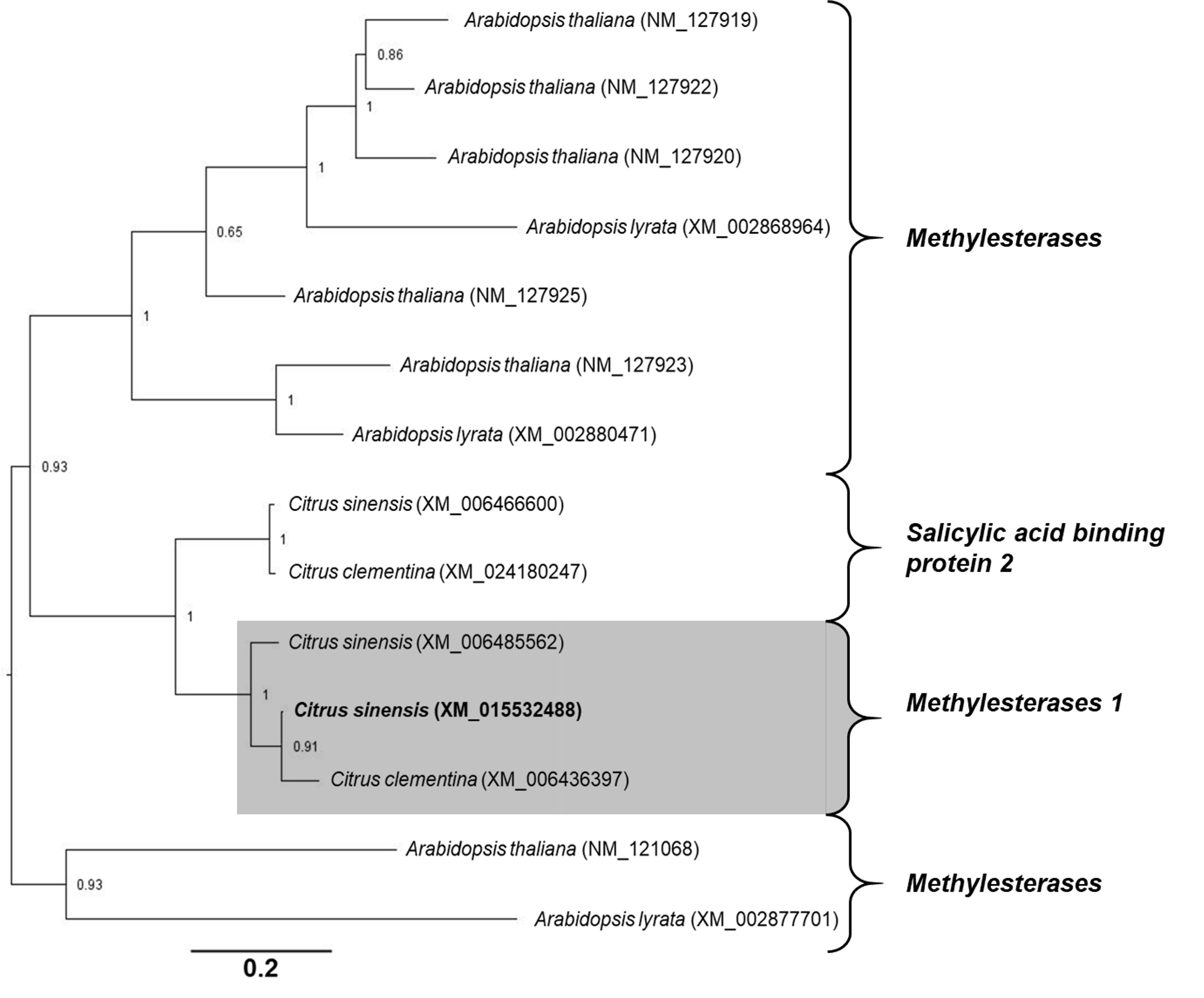

Supplement: Supplementary file 3 — Additional file 3 : Figure S3. Phylogenetic analysis of Methylesterases (MES). Bayesian analysis was performed using the ORF of MES1-like from C. sinensis and compared to homologous proteins from A. thaliana, A. lyrata and C. clementina. Numbers at the nodes denote posterior probabilities. The reference bar indicates the distance (number of amino acid substitutions per site). [file 12870_2019_2114_MOESM3_ESM.jpg]

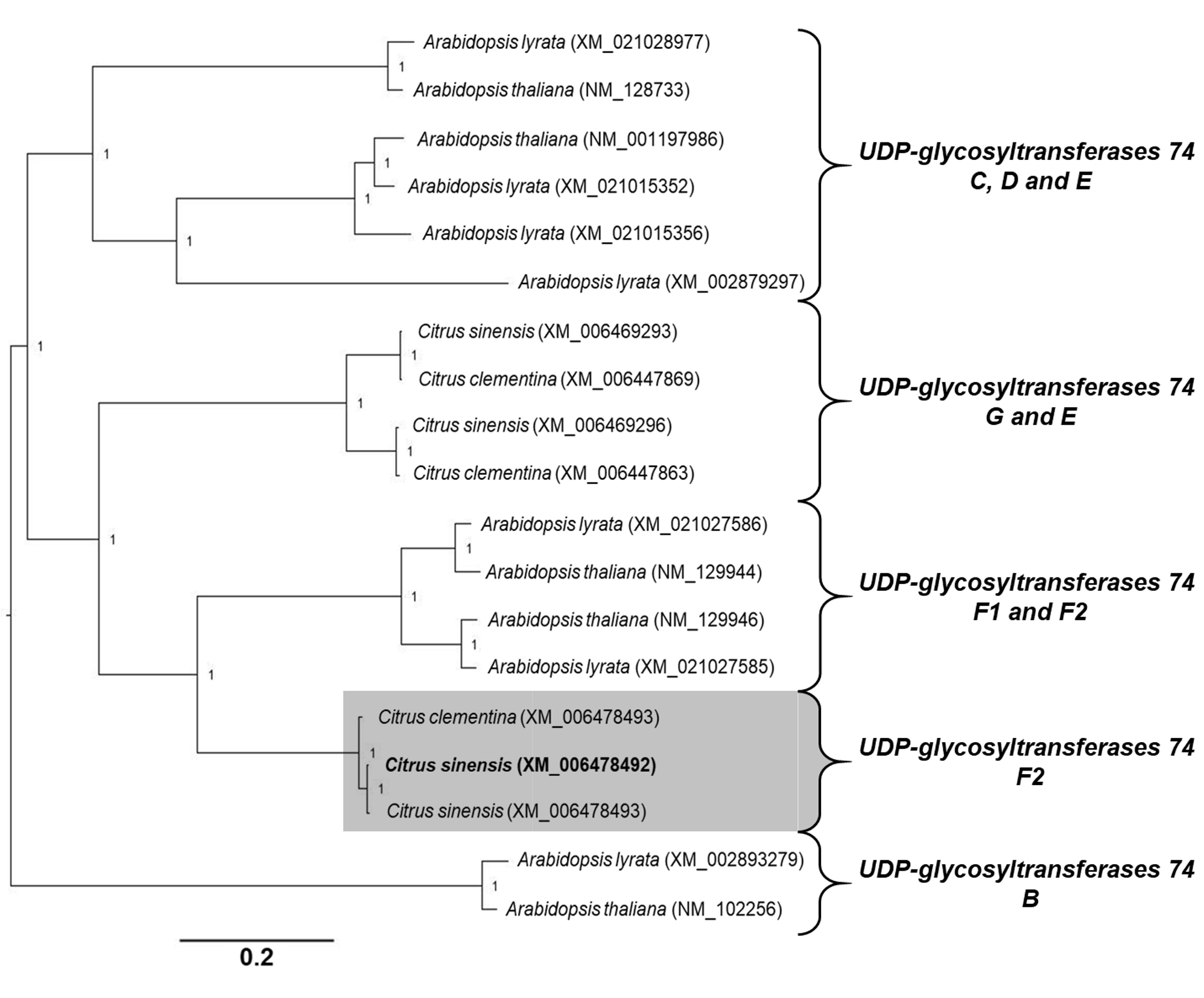

Supplement: Supplementary file 4 — Additional file 4: Figure S4. Phylogenetic analysis of UDP-glycosyltransferases 74 (UGT74). Bayesian analysis was performed using the ORF of UGT74F2-like from C. sinensis compared to and homologous proteins from A. thaliana, A. lyrata and C. clementina. Numbers at the nodes denote posterior probabilities. The reference bar indicates the distance (number of amino acid substitutions per site). [file 12870_2019_2114_MOESM4_ESM.jpg]

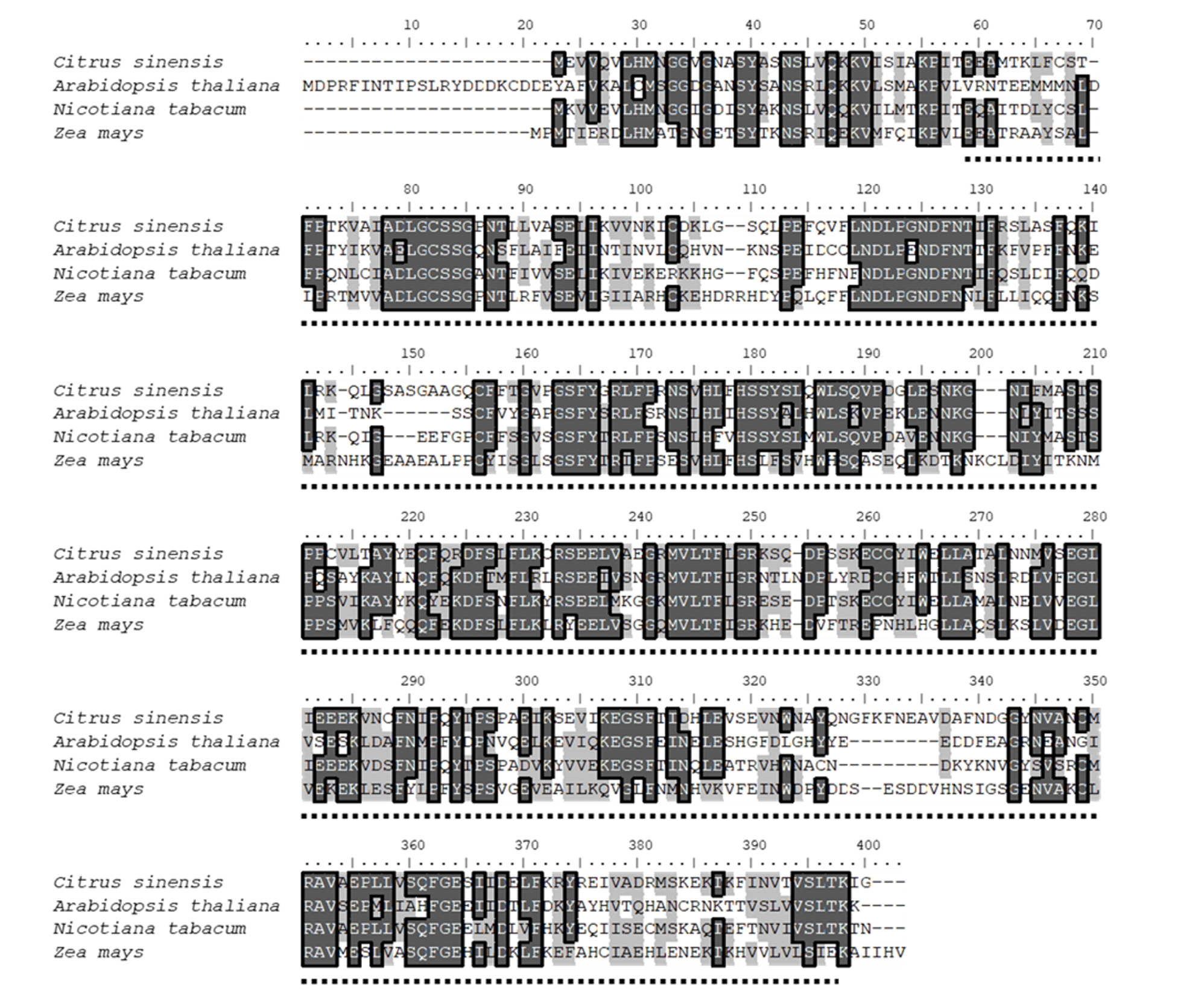

Supplement: Supplementary file 5 — Additional file 5: Figure S5. Alignment of S-adenosyl-L-methionine-dependent methyltransferase. Salicylate carboxymethyltransferase from C. sinensis was aligned with one species from each plant order (Solanales, Brassicales and Poales). Solanales represented by Nicotiana tabacum, Brassicales by Arabidopsis thaliana, and Poales by Zea mays. The Methyltransf_7 domain (pfam03492) found within these sequences is indicated by an underlined round dot line. [file 12870_2019_2114_MOESM5_ESM.jpg]

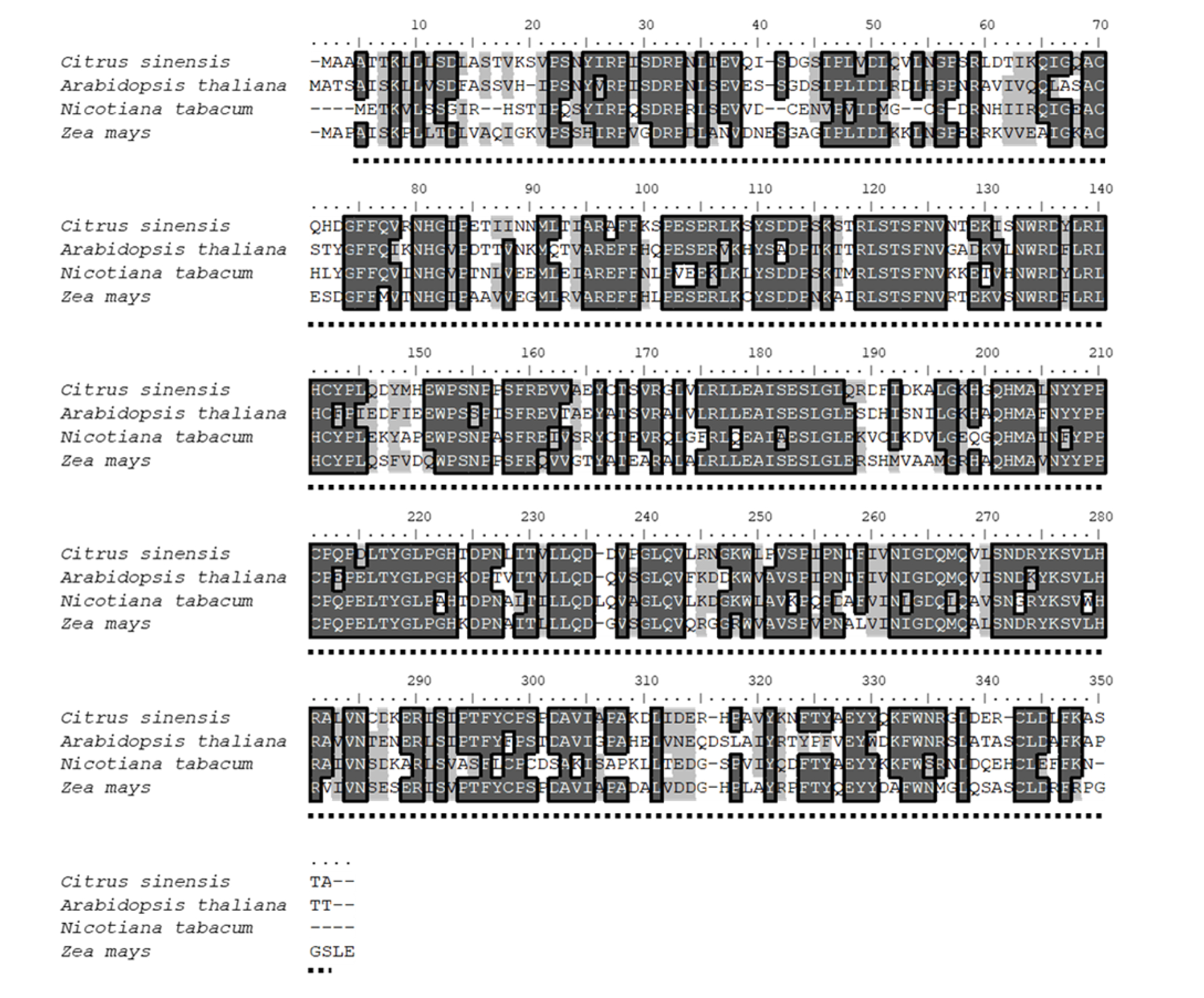

Supplement: Supplementary file 6 — Additional file 6: Figure S6. Amino acid alignment of DMR6-like oxygenase 1. DMR6-like oxygenase 1 from C. sinensis was aligned with one species from each plant order (Solanales, Brassicales and Poales). Solanales represented by Nicotiana tabacum, Brassicales by Arabidopsis thaliana, and Poales by Zea mays. The Oxidoreductase domain (PLN02912) found within these sequences was featured using an underline round dot line. [file 12870_2019_2114_MOESM6_ESM.jpg]

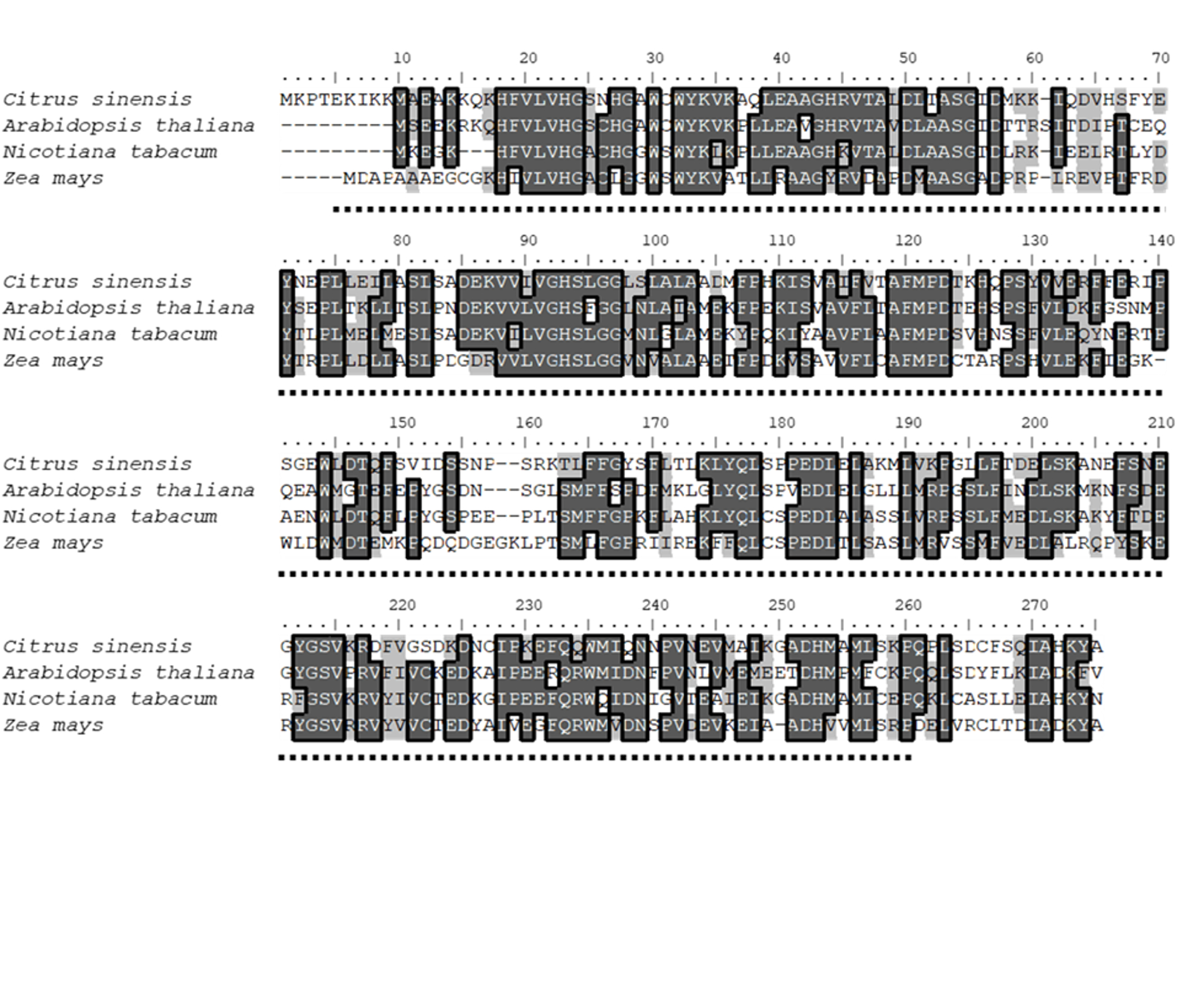

Supplement: Supplementary file 7 — Additional file 7: Figure S7. Alignment of Methylesterases. Methylesterase1-like from C. sinensis was aligned with one species from each plant order (Solanales, Brassicales and Poales). Solanales represented by Nicotiana tabacum, Brassicales by Arabidopsis thaliana, and Poales by Zea mays. The Methyl indole-3-acetate methyltransferase domain (PLN02211) found within these sequences is indicated by underlined round dot line. [file 12870_2019_2114_MOESM7_ESM.jpg]

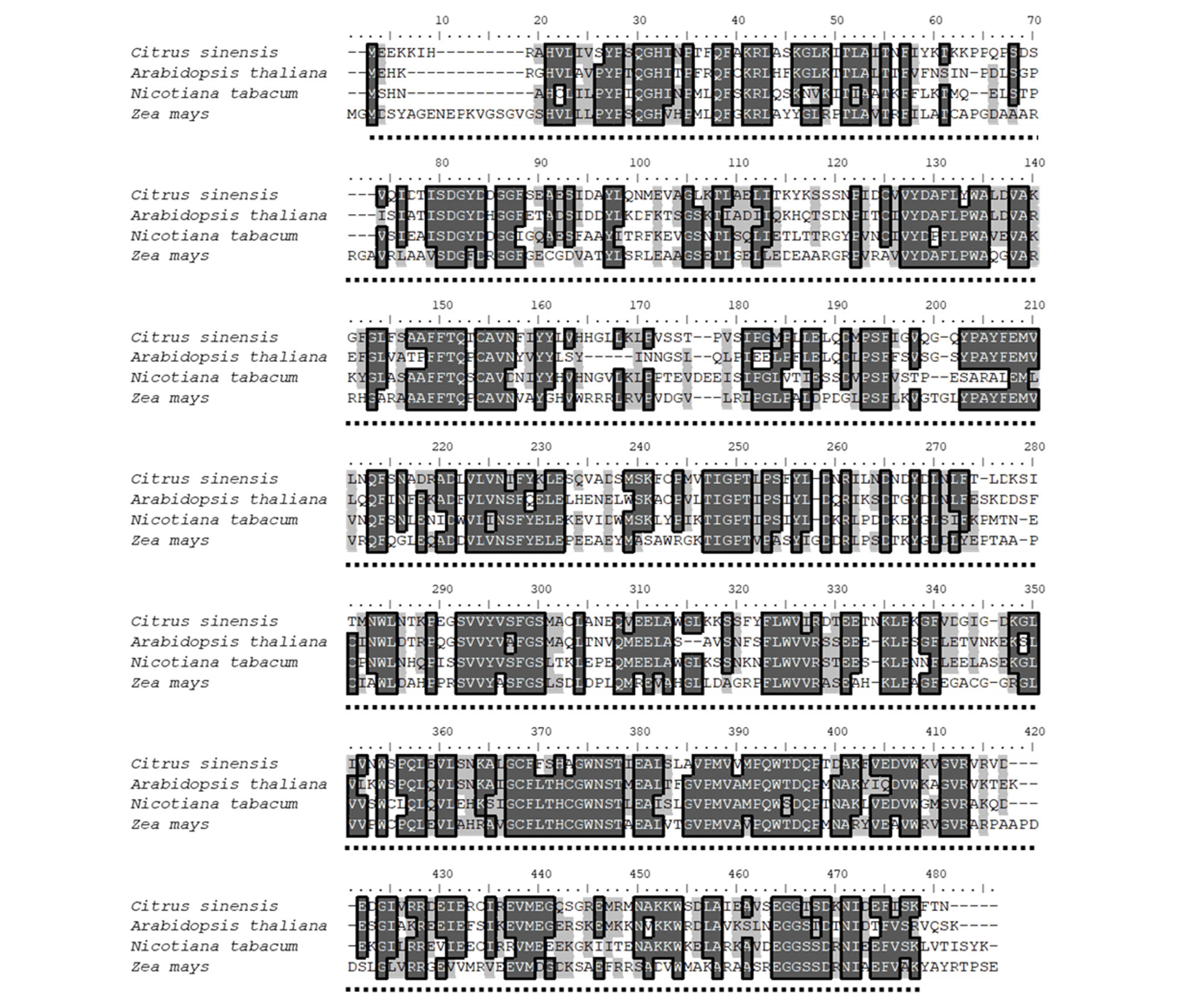

Supplement: Supplementary file 8 — Additional file 8: Figure S8. Alignment of UDP-Glycosyltransferase 74F2. UGT74F2 from C. sinensis was aligned with one species from each plant order (Solanales, Brassicales and Poales). Solanales represented by Nicotiana tabacum, Brassicales by Arabidopsis thaliana, and Poales by Zea mays. The Glycosyltransferase family 1 domain (cl10013) found within these sequences is indicated by underlined round dot line. [file 12870_2019_2114_MOESM8_ESM.jpg]

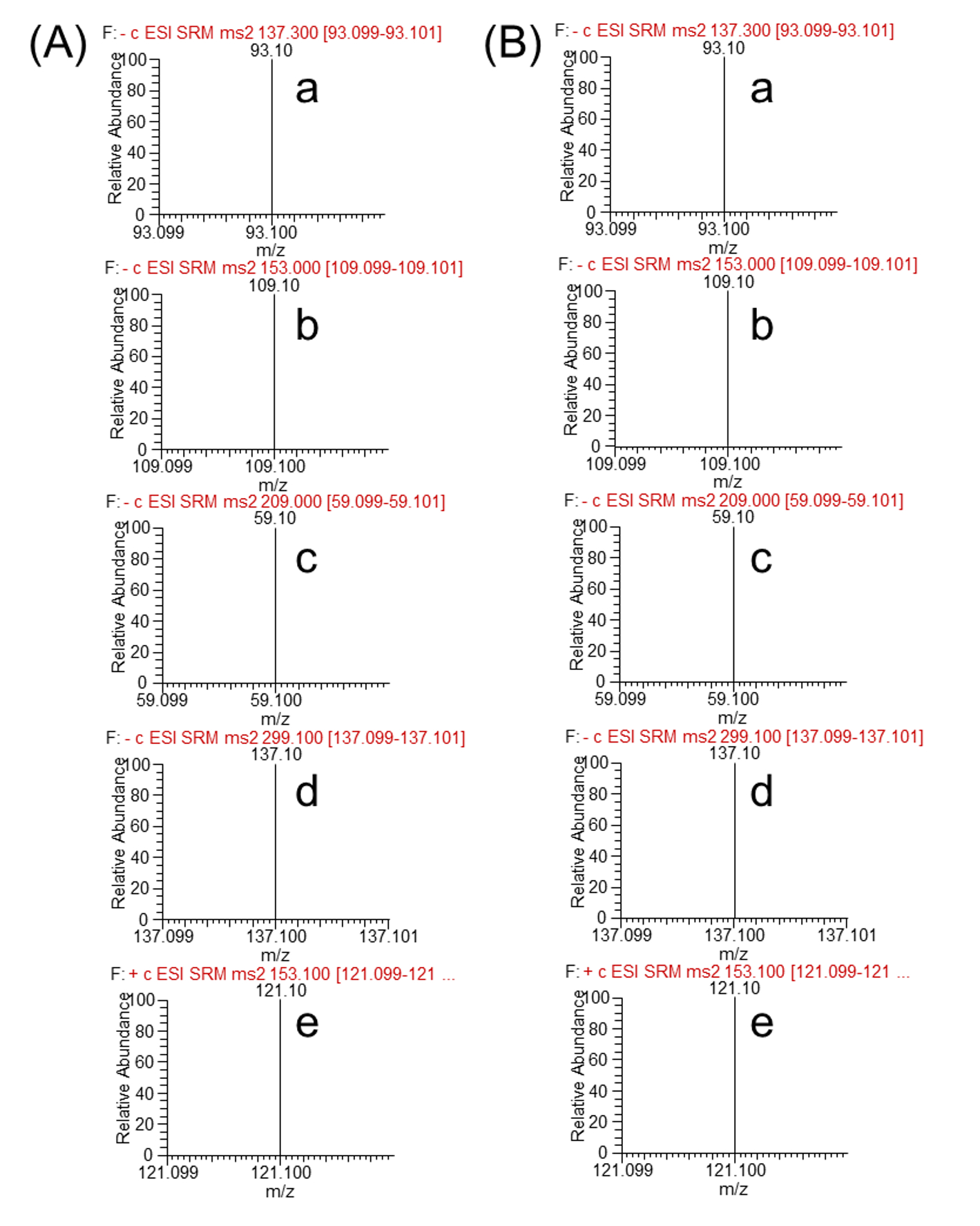

Supplement: Supplementary file 9 — Additional file 9: Figure S9. SRM spectra of (A) and (B) sample extract: a. salicylic acid (SA), b. 2,3-dihydroxybenzoic acid (2,3-DHBA), c. jasmonic acid (JA), d. salicylic acid 2-O-β-D-glucoside (SAG), and e. methyl salicylate (MeSA). [file 12870_2019_2114_MOESM9_ESM.jpg]

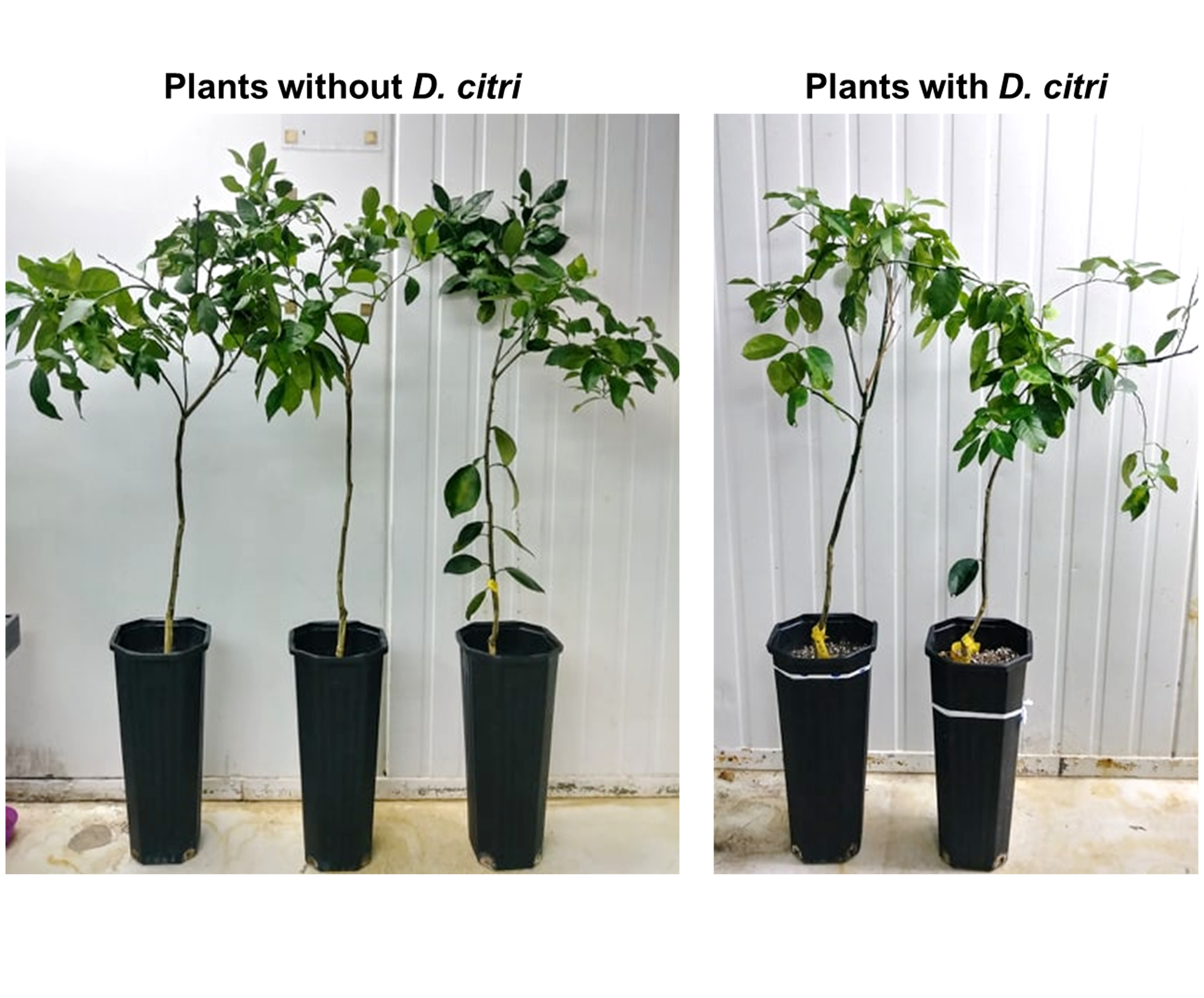

Supplement: Supplementary file 10 — Additional file 10: Figure S10. Representative image of C. sinensis plants. Left panel shows representative control plants after 150 days. Right panel shows plants exposed to insect feeding for 150 days. [file 12870_2019_2114_MOESM10_ESM.jpg]
